# Genotypic Diversity Analysis of *Mycobacterium tuberculosis* Strains Collected from Beijing in 2009, Using Spoligotyping and VNTR Typing

**DOI:** 10.1371/journal.pone.0106787

**Published:** 2014-09-19

**Authors:** Yi Liu, Miao Tian, Xueke Wang, Rongrong Wei, Qing Xing, Tizhuang Ma, Xiaoying Jiang, Wensheng Li, Zhiguo Zhang, Yu Xue, Xuxia Zhang, Wei Wang, Tao Wang, Feng Hong, Junjie Zhang, Sumin Wang, Chuanyou Li

**Affiliations:** 1 The Key Laboratory for Cell Proliferation and Regulation Biology, Ministry of Education, College of Life Sciences, Beijing Normal University, Haidian District, Beijing, China; 2 Department of Bacteriology and Immunology, Beijing Key Laboratory on Drug-resistant Tuberculosis Research, Beijing Tuberculosis and Thoracic Tumor Research Institute/Beijing Chest Hospital, Capital Medical University, Tongzhou District, Beijing, PR China; 3 Central Laboratory, Beijing Research Institute for Tuberculosis Control, Xicheng District, Beijing, PR China; 4 Clinical Center on TB, China CDC, Beijing Tuberculosis and Thoracic Tumor Research Institute/Beijing Chest Hospital, Capital Medical University, Tongzhou District, Beijing, PR China; 5 Beijing Changping Center for Tuberculosis Control and Prevention, Changping District, Beijing, PR China; Queen Mary Hospital, the University of Hong Kong, Hong Kong

## Abstract

**Background:**

Tuberculosis (TB) is a serious problem in China. While there have been some studies on the nationwide genotyping of *Mycobacterium tuberculosis* (*M. tuberculosis*), there has been little detailed research in Beijing, the capital of China, which has a huge population. Here, *M. tuberculosis* clinical strains collected in Beijing during 2009 were genotyped by classical methods.

**Methodology/Principal Findings:**

Our aim was to analyze the genetic diversity of *M. tuberculosis* strains within the Beijing metropolitan area. We characterized these strains using two standard methods, spoligotyping (n = 1585) and variable number of tandem repeat (VNTR) typing (n = 1053). We found that the most prominent genotype was Beijing family genotype. Other genotypes included the MANU, T and H families etc. Spoligotyping resulted in 137 type patterns, included 101 unclustered strains and 1484 strains clustered into 36 clusters. In VNTR typing analysis, we selected 12-locus (QUB-11b, MIRU10, Mtub21, MIRU 23, MIRU39, MIRU16, MIRU40, MIRU31, Mtub24, Mtub04, MIRU20, and QUB-4156c) and named it 12-locus (BJ) VNTR. VNTR resulted in 869 type patterns, included 796 unclustered strains and 257 strains clustered into 73 clusters. It has almost equal discriminatory power to the 24-locus VNTR.

**Conclusions/Significance:**

Our study provides a detailed characterization of the genotypic diversity of *M. tuberculosis* in Beijing. Combining spoligotyping and VNTR typing to study the genotyping of *M. tuberculosis* gave superior results than when these techniques were used separately. Our results indicated that Beijing family strains were still the most prevalent *M. tuberculosis* in Beijing. Moreover, VNTR typing analyzing of *M. tuberculosis* strains in Beijing was successfully accomplished using 12-locus (BJ) VNTR. This method used for strains genotyping from the Beijing metropolitan area was comparable. This study will not only provide TB researchers with valuable information for related studies, but also provides guidance for the prevention and control of TB in Beijing.

## Introduction

Tuberculosis (TB), caused by *Mycobacterium tuberculosis* (*M*. *tuberculosis*), is one of the major causes of death in the world today [1]. It is well known that China was the one of the 22 TB high-burden countries in the world [1]. TB is a reemerging infectious disease and a substantial public health problem in Beijing [2], [3], the capital of the People’s Republic of China, a megacity containing many districts with large populations and high population mobility. It has been reported that the prevalence and incidence of TB is gradually becoming higher in Beijing [4], thus the prevention and control of TB is a great challenge for this city.

Although Beijing has been thought of as the headstream of the Beijing family strains of *M. tuberculosis*, only a few studies have been conducted on strains collected in the Beijing area. Jia et al. investigated the distribution and magnitude of TB cases in permanent residents and migrant populations of Beijing between 2000 and 2006 [5], but they did not carry out genotyping analyses. Wan et al. [6] also performed studies on the diversity of *M. tuberculosis* in Beijing using strains collected randomly at the Beijing TB control and cure Institutes, and TB hospitals during the period 2005 to 2007, but only selected a fraction of the strains acquired from Beijing city, and not all strains. The strains chosen were not sufficiently representative to reveal the true prevalence and genotypic diversity of TB in Beijing. As the molecular epidemiology of TB in Beijing has not previously been well studied, we designed a study to examine the genetic diversity of TB strains by genotyping strains collected in 2009 from different areas of Beijing.

Spacer oligonucleotide typing (Spoligotyping) is a rapid and convenient genotyping method that is well suited for the identification of Beijing family *M. tuberculosis* strains [5]. However, it has a fairly low discriminatory power [7]. Variable number of tandem repeat (VNTR) genotyping is technically easier and possesses high discriminatory power and can therefore complement spoligotyping. Therefore, we combined these two methods together in this study. Moreover, its digital results are easy to be compared between different laboratories [8], [9]. Studying the molecular epidemiology of *M. tuberculosis* has been proven to be a useful tool for TB study and control, as it can be used to tracking transmission chains and detect suspected outbreaks [10]. In 2008, Jiao established in 2008 that classical VNTR methods can differentiate Beijing strains from other strains [11].

In this study, we have combined spoligotyping and VNTR to map the genotypic and molecular epidemiology of TB strains collected in Beijing during 2009. A collection of 1585 representative *M. tuberculosis* strains isolates was first genotyped by spoligotyping, and 1053 *M. tuberculosis* strains isolates were then genotyped by VNTR. We found that the most prominent *M. tuberculosis* family in Beijing in 2009 was the Beijing family; other families included the MANU2, T1, T2 and H families et al. VNTR typing analysis selected 12 loci as the VNTR genotype locus applied to Beijing strains. Our findings should contribute to the design of more efficient strategies for TB prevention and control in Beijing.

## Results

### Spoligotyping-based diversity of *M. tuberculosis* strains collected from the Beijing metropolitan area

**Figure 1:**
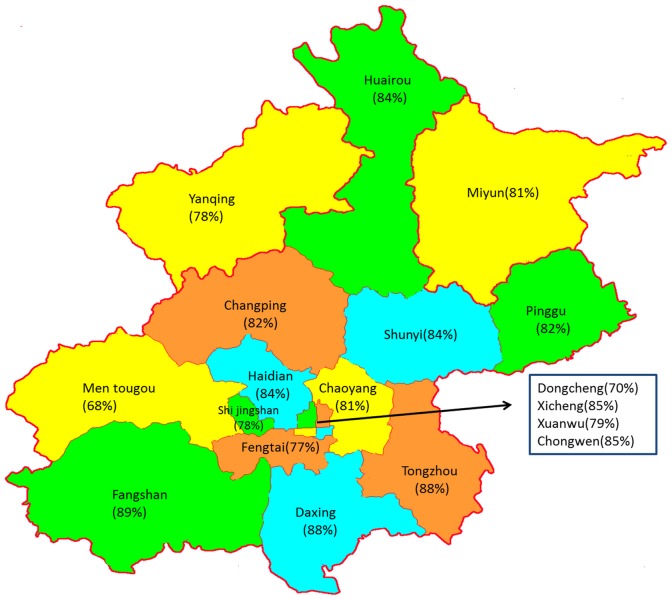
The ratio of Beijing genotype strains accounted for in each district of Beijing in 2009.

To demonstrate spoligotype diversity among *M. tuberculosis* strains in the Beijing metropolitan area, we collected 1585 strains from different districts of Beijing (Figure S1) during 2009. The strains number of four districts of Chaoyang, Haidian, Xicheng and Xuanwu occupied more than 1/2 of the total strains. Spoligotyping data profiles of the all strains isolates can be seen in Table S4. Thirteen hundred (82%) strains were Beijing family strains, and 285(18.1%) strains belonging to non-Beijing family (Table 1). In all Beijing lineage strains, 1225 strains (77.3%) were typical Beijing genotype strains, and 75 (4.7%) were atypical Beijing genotype strains. The percentage of strains with a Beijing genotype exceeded 67% in all districts (Figure 1). Of the non-Beijing strains, 105 (6.6% of all strains) belonged to the poorly-defined T lineage, 69 (4.4%) being from the T1 (SIT53) lineage, 24 (1.6%) from the T2 (SIT52) lineage, 10 (0.6%) from the T3 (SIT37) lineage and 2 from the T4 lineage. In addition, 76 (4.8%) strains were from the MANU2 (SIT54) lineage, 12 (0.75%) from the U lineage and 84 (5.3%) were of an “Unknown” genotype (absent from the current global spoligotyping database: Spo1DB4). Two *Mycobacterium bovis* Bacillus Calmette-Guérin (BCG) strains, unique strains deserving careful follow-up study, were also found.

**Table 1:** Spoligotyping patterns result of *M. tuberculosis* strains collected from Beijing in 2009.

Year	No.$	No. (%)													
		Beijing families*		non-Beijing families#											
		Typical Beijing	Atypical Beijing	T1	T2	T3	T4	MANU2	H3	H4	U	S	CAS1- Delhi	BCG	Unknown
2009	1585	1225 (77.28%)	75 (4.73%)	69 (4.35%)	24 (1.51%)	10 (0.63%)	2	76 (4.79%)	3	1	12 (0.75%)	1	1	2	84 (5.29%)

$ The number of collected strains isolates.

*1300(82.01%) strains belonged to Beijing families.

# 285 strains (include 84 unknown strains) belonged to non-Beijing families.

The numbers and frequencies of strains collected in Beijing in 2009, along with their spoligotypes were listed in Table 2. 1484 strains were been formed 36 clusters, only 101 strains were not been clustered. Thus the ratio of cluster of spoligotyping method was fairly high. As summarized in Table 3, spoligotyping clustered 1431(90%) strains into 8 maximum clusters. Among these 8 types, SIT1, SIT190, SIT265 and SIT269 were members of the Beijing family lineage, and included 1225 (77.3%), 23 (1.45%), 20(1.26%) and 11 (0.69%) of the strains, respectively. SIT37 (T3 lineage), SIT52 (T2 lineage), SIT53 (T1 lineage) and SIT54 (MANU2 lineage) belonged to the non-Beijing family. More data were summarized in Table S1, we list all spoligotyping shared types (n = 1585 strains) appeared in this research.

**Table 2:** Numbers and frequencies of strains clustered assorted by spoligotyping from 2009 years in Beijing.

Spoligotyping Parameter	Value
No. of strains studied	1585
No. of clusters	36
No. of unknown spoligotype	84
Mean No. of strains per cluster	41.22
No.(%) of clustered strains	1484 (93.62)
No.(%) of unclustered strains	101 (6.37)

**Table 3:** The description of these clusters containing the maximum 8 clusters of *M. tuberculosis* collected from Beijing in this study.

Sequence number	SIT *(Clade) Octal number spoligotype description	Number(%#) in study
1	1 (Typical Beijing) 000000000003771	1225(77.28)
2	37(T3) 777777777760700	6(0.26%)
3	52(T2) 777777777760731	24(1.51%)
4	53(T1) 777777777760771	69(4.35%)
5	54(MANU2) 777777777763771	76(4.79%)
6	190(Beijing like) 000000000003731	23(1.45%)
7	265(Beijing like) 000000000003371	20(1.26%)
8	269(Beijing like) 000000000000771	11(0.69%)

*spoligotype international type, SIT number from SpoIDB4.0.

# Represents the percentage of strains with a common SIT among all strains in this study.

We also analyzed the relationship between the Beijing family and sex, age (≤45 and >45), and the household address of patients (this city or other areas). We found a statistically significant difference (P = 0.01<0.05) in sex (Table S2), of which the proportion of male infections with Beijing family genotype strains higher than the proportion of female infections. There were no statistically significant associations with age and household registration.

The population clad structure of TB strains in Beijing, based on major *M. tuberculosis* complex (MTBC) clades, was as follows: East Asian (Beijing) (82%), Euro-American (9.5%), Indo-Oceanic (8.3%), *Mycobacterium bovis* BCG (0.1%) and unknown (0.1%) (Table S3). We also investigated the proportion of modern and ancient lineages [12], [13] among these groups. In this study, modern lineages,including Beijing/T/H lineages, accounted for 88.8% of all strains. Ancestral lineages, including the MANU/U/S and CAS lineages, accounted for only 11.2% of all strains. These results indicate modern lineage strains were the most epidemiological strains in the Beijing metropolitan area.

### The selection of VNTR loci and the result of VNTR genotyping

To determine whether different VNTR loci possess discrepant discriminatory power when applied to these strains, we genotyped 1053 of the TB strains collected in Beijing during 2009 using the VNTR method (Table S4). As a preliminary experiment, we first selected 13 loci (QUB-11b, MIRU10, Mtub21, MIRU39, MIRU16, MIRU40, MIRU31, Mtub24, Mtub04, QUB-4156c, Mtub39, Rv-2372, and Qub-15) to genotype the 465 strains collected during 2008 (data not shown). Results indicated that several loci were not suitable for genotyping as their, PCR products were not obtained consistently despite repeated attempts. We therefore eliminated 4 genotype loci (Mtub24, Mtub04, Qub-15, and Rv-2372), and added 3 new loci (MIRU 23, ETR-A and Mtub30) to study the strains collected in 2009 by comparing allelic diversity of different VNTR loci from different areas, bringing the total number of genotype loci included in this study to 12 (Table 4). QUB-4156c (VNTR 4156), MIRU23 (VNTR 2531), MIRU20 (VNTR 3007) and MIRU39 (VNTR 4348) presented low levels of allelic diversity but were useful for the confirmation of different lineages [14] and were thus included in the analysis.

**Table 4:** The Hunter-Gaston discriminatory index of the 12 VNTR loci in *M. tuberculosis* strains from Beijing in 2009.

Order	VNTR# locus	VNTR alias	Number of alleles	Range of repeats	Allelic diversity (h*) for		
					Beijing family (n = 884)	non-Beijing family (n = 169)	All strains (n = 1053)
1	2163	QUB-11b	14	1–10	0.7262	0.7687	0.7637
2	1955	Mtub21	9	1–9	0.6881	0.7339	0.7196
3	4348	MIRU39	7	2–9	0.6848	0.5691	0.7172
4	1644	MIRU16	6	0–8	0.6923	0.7333	0.6962
5	3192	MIRU31	6	0–9	0.6544	0.7394	0.6518
6	0960	MIRU10	6	0–7	0.4990	0.7312	0.5478
7	0802	MIRU40	5	1–7	0.4007	0.5987	0.4725
8	2165	ETR A	5	0–6	0.3760	0.5749	0.4261
9	4156	QUB-4156c	8	0–7,11	0.2867	0.2938	0.2992
10	2401	Mtub30	5	1–10	0.1727	0.5409	0.2459
11	3690	Mtub39	6	1–7	0.2300	0.4136	0.2723
12	2531	MIRU23	7	0–7	0.0443	0.1386	0.0561

*Hunter-Gaston discriminatory index.

# Variable number tandem repeat.

The 12-locus (BJ) VNTR method differentiated the 1053 strains into 869 genotypes (Table 5). A total of 796 strains had unique patterns and the remaining 257 formed 73 clusters (2 to 20 strains per cluster). The allelic diversity for the 1053 strains of each VNTR locus was estimated using the Hunter-Gaston discriminatory index (HGDI) (Table 4). The discriminatory power of 5 loci (QUB-11b, Mtub21, MIRU39, MIRU16, and MIRU31) exceeded 0.5 and these were regarded as highly discriminatory. Three loci (ETRA, MIRU10 and MIRU40) showed moderately high discriminatory (0.3 to 0.5). Other loci (QUB-4156c, Mtub30, Mtub39 and MIRU23) were found to be less polymorphic, with HGDI within the range of 0.04 to 0.3. Of the 12 VNTR loci, QUB-11b had the greatest allelic diversity and the highest discriminatory power for all genotypes including Beijing genotype strains. All results can be seen in Table 4.

**Table 5:** Different discriminatory power of different typing methods used in this study.

Method	Beijing Family % (No.)	No. of type patterns	No. of unique strains	No. of clustered strains	No. of clusters	Cluster size (No. of strains)	Clustering rate (%)	Maximum No. of strainsin a cluster	Allelic diversity (h*) for		
									All strains	Beijing family	Other strains
Spoligotyping (1585)	82%(1300)	137	101	1484	36	2–1225	93.6	1225	0.3999	0.1110	0.9177
Spoligotyping (1053)	83.8%(884)	105	80	973	25	2–830	92.4	830	0.3759	0.1190	0.9234
12-locus VNTR(1053)	83.8%(884)	869	796	257	73	2–20	25.84	20	0.9990	0.9984	0.9994
VNTR and Spoligotyping(1053)	83.8%(884)	899	847	206	52	2–16	19.56	16	0.9994	0.9989	0.9996

*Hunter-Gaston discriminatory index.

### Comparison of spoligotyping and VNTR genotyping methods

Spoligotyping and VNTR genotyping results were summarized in Table 5 which showed the discriminatory power of the two genotyping methods. Spoligotyping (n = 1585) had a low discriminatory power (HGDI = 0.399), especially when applied to Beijing family strains (HGDI = 0.111) (Table 5); among 115 different types, spoligotyping identified 101 unique strains (8.4%) and grouped 1484 (91.6%) strains into 17 clusters, giving a clustering rate of 93.6%. The largest cluster was assigned to the typical Beijing genotype and included 1225 strains. Compared to spoligotyping, the original set of 12-locus (BJ) VNTR alone (n = 1053) identified 796 unique strains and clustered 257 strains into 73 clusters, giving a significantly higher discriminatory power (HGDI = 0.9984). When spoligotyping and 12-VNTR methods were used together (n = 1053), the discriminatory power increased, giving an HGDI value of 0.9989, and the clustering rate was reduced to 19.6% (Table 5). This study showed that spoligotyping and VNTR are appropriate methods for constructing primary genotype libraries of these strains, and the combination of these methods provides high discriminatory power.

### Comparison of the discriminatory power of kinds of VNTR loci

In this study, we did not use 15-locus and 24-locus VNTR primer sets [9], because these loci were not always suited to strains from the Beijing district. We found that the allelic diversity of the VNTR loci varied significantly used to Beijing family isolates in different countries and districts when the HGDIs of these loci were compared with those reported from other areas (Table 6).

**Table 6:** Allelic diversity (h*) of different VNTR loci in Beijing family isolates from different areas.

Order	VNTR locus	VNTR alias	Beijing, China(this study)	Shanghai, China [16]	Heilongjiang, China [40]	Hong kong, China [41]	Japan [15]	Russia [42]
1	2163	QUB-11b	0.7262	0.6548	0.644	–	0.815	0.205
2	1955	Mtub21	0.6881	0.5231	0.396	–	0.598	0.330
3	4348	MIRU39	0.6848	0.2856	0.290	0.320	0.156	0.000
4	1644	MIRU16	0.6923	0.2423	0.200	0.058	0.258	0.082
5	3192	MIRU31	0.6544	0.2461	0.599	None	0.270	0.160
6	0960	MIRU10	0.4990	0.1952	0.154	0.377	0.431	0.082
7	0802	MIRU40	0.4007	0.1471	0.292	0.196	0.229	0.122
8	2165	ETR A	0.3760	0.0308	0.238	0.201	0.223	0.158
9	4156	QUB-4156c	0.2867	0.4923	0.107	–	–	–
10	2401	Mtub30	0.1727	0.0909	0.133	–	0.379	0.042
11	3690	Mtub39	0.2300	0.0606	0.174	–	0.215	0.000
12	2531	MIRU23	0.0443	0.0606	–	–	0.158	0.000

*Hunter-Gaston discriminatory index.

To verify the discriminatory power of each locus and the cumulative discriminatory power of all 12-locus (BJ), we calculated the discriminatory power of the loci listed in Table 7 to get an indication of allelic diversity and cumulative HGDI discriminatory power. The respective discriminatory powers of 4 VNTRs methods, (15-locus (Supply) [9], 24-locus (Supply) [9], [13], 12-locus (JATA) [15], and 16-locus (Gao) [16]) were compared (Table 8). The cumulative HGDI discriminatory power of these 12-locus (BJ) VNTR reached 0.9990 when applied to all strains, 0.9994 when applied to non-Beijing family and 0.9984 when applied to Beijing family (Table 7 & Table 8). These results show that our VNTR system is superior to the reported 15-locus VNTR and has almost equal discriminatory power to the 24-locus VNTR, 12-locus (JATA) and 16-locus (Gao) (Table 8), especially when applied to Beijing family strains, although it only uses 12 loci. Therefore, our 12-locus (BJ) VNTR method and the methods used for strains from the Beijing metropolitan area are comparable.

**Table 7:** Allelic diversity of the 12 VNTR loci in *M. tuberculosis* strains from Beijing 2009.

Order	VNTR locus	VNTR alias	Beijing family (n = 884 )		non-Beijing family (n = 169)		All strains(n = 1053 )	
			HGDI(Individual locus)	HGDI* (cumulative)	HGDI(Individual locus)	HGDI (cumulative)	HGDI(Individual locus)	HGDI (cumulative)
1	2163	QUB-11b	0.7262	0.7262	0.7687	0.7687	0.7637	0.7637
2	1955	Mtub21	0.6881	0.8382	0.7339	0.8871	0.7196	0.7974
3	4348	MIRU39	0.6848	0.9479	0.5691	0.9765	0.7172	0.9528
4	1644	MIRU16	0.6923	0.9766	0.7333	0.9904	0.6962	0.9786
5	3192	MIRU31	0.6544	0.9926	0.7394	0.9972	0.6518	0.9927
6	0960	MIRU10	0.4990	0.9958	0.7312	0.9976	0.5478	0.9940
7	0802	MIRU40	0.4007	0.9968	0.5987	0.9981	0.4725	0.9961
8	2165	ETR A	0.3760	0.9972	0.5749	0.9985	0.4261	0.9969
9	4156	QUB-4156c	0.2867	0.9975	0.2938	0.9988	0.2992	0.9976
10	2401	Mtub30	0.1727	0.9976	0.5409	0.9990	0.2459	0.9983
11	3690	Mtub39	0.2300	0.9983	0.4136	0.9993	0.2723	0.9989
12	2531	MIRU23	0.0443	0.9984	0.1386	0.9994	0.0561	0.9990

*Hunter-Gaston discriminatory index.

**Table 8:** The comparison of the discriminatory power combination of different VNTR loci in different country.

Typing methods	All strains				
	HGDI*	No. of types	No. of unique types	No. of clusters	Percentage of clustering
15-locus(Supply)	0.990	291	269	22	17.2
24-locus (Supply)	0.999	303	287	16	11.7
12-locus (JATA)	0.999	302	284	18	12.6
16-locus (Gao)	0.9983	183	–	27	14.9
12-locus (this paper)	0.999	869	796	73	25.84

*Hunter-Gaston discriminatory index.

### Distribution of clustering shown by the minimum spanning tree using spoligotype and VNTR data

Another way to represent the strains graphically is to construct a minimum spanning tree, which shows the clustering of TB strains. To show the major clusters and map their genetic links map, we constructed minimum spanning trees using BioNumerics software.

**Figure 2:**
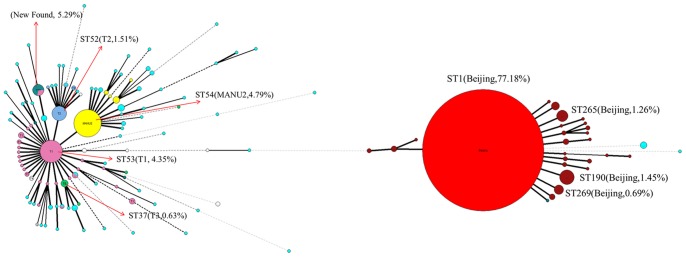
Minimum spanning tree showing the clustering by Spoligotyping of 1585* M. tuberculosis* strains of Beijing. Each nodal point represents a particular spoligotype, and the size of nodal point is relative to the number of strains with that spoligotype. The percentage represents the proportion of each spoligotype. The annotations in the figure were the 8 most frequent spoligotypes.

Minimum spanning trees were first generated by using spoligotype data (Figure 2). Each nodal point represented a particular spoligotype type, and the size of each nodal point was related to the number of strains within that spoligotype. As shown in Figure 2, the biggest cluster represents the Beijing genotype, but clusters of atypical Beijing genotype were also seen. The Beijing and non-Beijing family clusters were divided into two large groups. Annotations in the figure represented the 8 most frequent spoligotypes found in the Beijing municipality. The largest cluster (ST1) corresponded to the Beijing strains, as confirmed by spoligotyping, and three clusters (ST190, ST265 and ST269) showed the signature of strains belonging to the Beijing-like strains. According to the minimum spanning tree, the new SIT type was more similar to non-Beijing spoligotypes.

Other clusters showed mutually recognizable with spoligotype signatures. However, for some strains, there was no concordance with the spoligotyping results. In particular, 10 newly-identified strains with a spoligotype profile corresponding to MANU2 were clustered with Beijing strains. These discordant findings may result from mixed strains.

**Figure 3:**
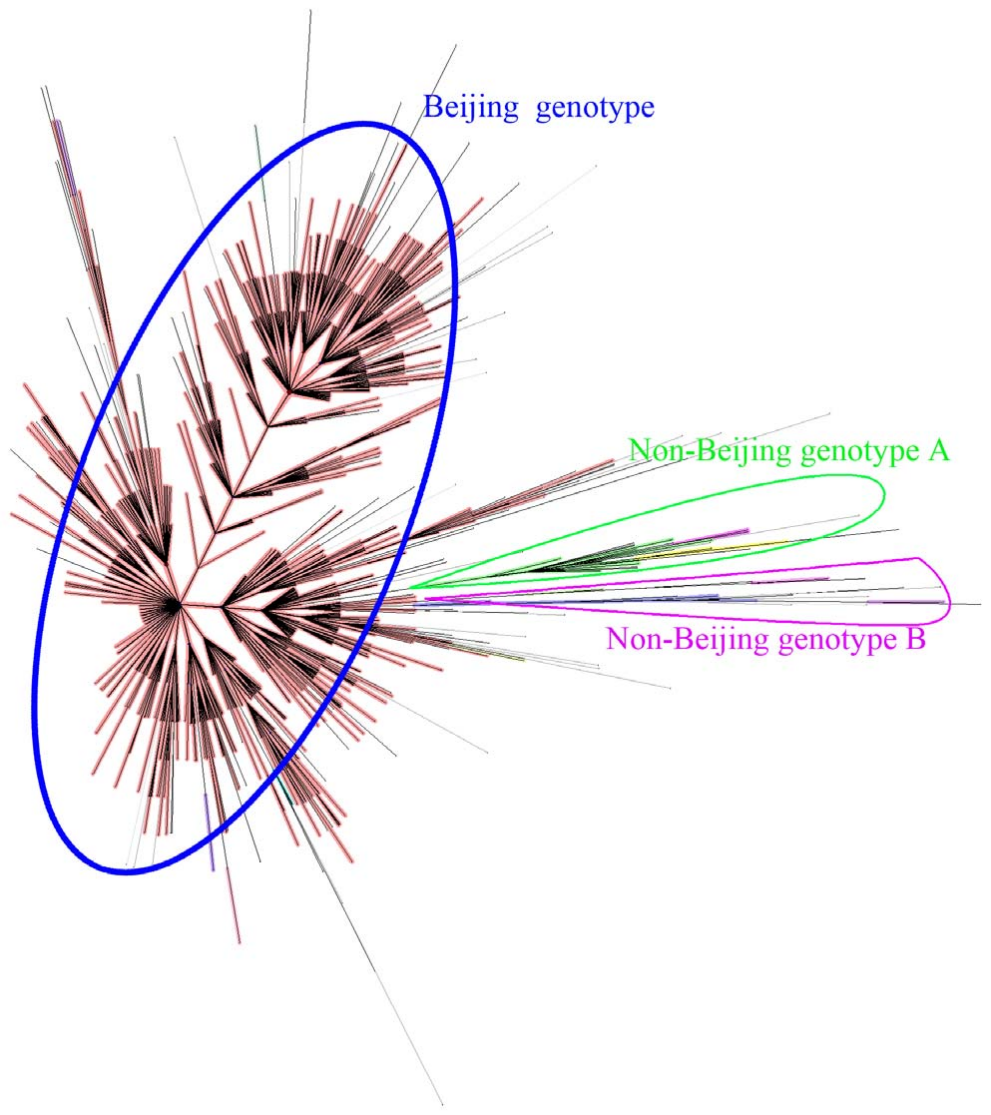
Minimum spanning tree showing the clustering by 12-locus (BJ) VNTR of 1053 *M. tuberculosis* strains of Beijing. Different clusters are shown in different colors; the largest light pink group represents Beijing family isolates, whereas orange, green and blue represent the non-Beijing family groups.

In Figure 3, the minimum spanning tree showed the clustering of 1053 Beijing strains using the VNTR typing method; different clusters were shown in different colors. From the figure clearly shows that the polymorphism of these strains was very high, and that this VNTR typing method has high discriminatory power. Although it was possible to assign strains to different branches, it was difficult to discern the evolutionary subtype to which each strain belongs. Figure 3 shows that the Beijing family strains form the largest group (light red). Non-Beijing family groups were shown in orange, green, purple and blue etc. In this MST tree, Beijing family strains were separated from non-Beijing family strains. Cluster analysis show identified 73 clusters (2 to 20 strains per cluster). Almost all of the clustered strains belonged to Beijing family genotype; only two clusters contained 2 and 3 strains respectively that were from the MANU2 family.

## Discussion

TB is a serious problem globally, and its prevalence is reaching epidemic proportions in countries and districts with large and mobile populations, including many megacities. DNA fingerprinting technology is now a commonly used tool to detect and investigate outbreaks of *M. tuberculosis*. Particular *M. tuberculosis* genotypes have been found to be related to the risk of transmission. Previous studies [6], [16]–[18] have described the distribution of various genotypes of TB strains from many areas in China. However, a detailed analysis of the population structure of TB strains from the metropolitan area of Beijing, one of the largest cities in the world, with a high prevalence of TB, has not been conducted. Acquiring information on the genotype diversity of TB strains in Beijing and exploring their molecular characteristics is important for understanding and controlling the spread of TB.

To identify which strains of TB were prevalent strains occur in Beijing, we genotyped strains using spoligotyping and VNTR typing. Spoligotyping is a classical method used for TB genotype analysis, especially for the identification of Beijing family strains [19], although its discriminatory power is not high. VNTR has been used extensively around the world [20], and was recommended as a standard genotyping method. The characteristics of prevalent strains in different regions are easy to compare using the digital features available with VNTR genotyping.

A previous study in which we genotyped on clinical samples of Beijing TB strains collected from several areas of China provided us with baseline information on the distribution of dominant TB genotypes in China [17]. However, the strains selected in that study do not accurately represent the prevalence and epidemic trends of TB within metropolitan Beijing. Here, we genotyped 1565 strains collected from 18 districts and counties in Beijing. As shown in Table 1 and Table 5, 1300 (82%) of these strains from the Beijing district belonged to the Beijing family, a finding very similar to results reported by from the Beijing district other labs [21].

The prevalence of the Beijing family in Beijing (82%) is higher than in Hong Kong (70%) [22], Taiwan (44.4%) [23], Vietnam (54%) [24], Thailand (44%) [25]and Russia (44.5%) [26]. Although the Beijing family was the dominant genotypic family in the Beijing district, genotypic polymorphisms were also evident. In this study, non-Beijing family strains from the T1, T2, T3, T4, MANU2 and U families. Although modern lineages were the most epidemiological strains in the Beijing metropolitan area, but ancient lineages were also been found. It was interesting, that U and CAS1-Delhi types were detected in Beijing. It has previously been reported [13], [27] that the Middle East, Central, and Southern Asia are ‘high incidence’ areas for these strains. Hence, frequent movement of ethnic groups from the Xinjiang autonomous region may be a major factor explaining the increasing prevalence of these genotypes in Beijing. The T family genotype, one of the prevalent genotypes in Africa, Central, South America and Europe, was found in this study [28]. This result showed movement of groups from these regions may increase prevalence of these genotypes in Beijing. In addition, novel spoligotypes were identified in this study. It showed that the complexity of the Beijing metropolitan area strains source, which need more deep study.

The population structure of TB strains in Beijing consists of East Asian (Beijing), Euro-American, Indo-Oceanic, *Mycobacterium bovis* BCG and strains of unknown origin. The *Mycobacterium bovis* BCG strains deserve particular mentioned. The exact source of these strains is unknown. It is unclear if patients were really been infected by *Mycobacterium bovis* BCG or this was only a case of laboratory contamination. We will carry out a full investigation in the future.

We also found that there were statistically significant differences between the number of male and female patients infected by Beijing genotype strains, with the ratio of infected males being significantly higher than that for infected female. However, there were no statistically significant associations between the Beijing strain and age or household registration. These results differ slightly from other areas [29], but these data should give an accurate picture of Beijing TB strains. Our results illustrated the fact that research on strains in the Beijing area must be based on a large-scale study in order to obtain real and accurate results.

In particular, because PCR products of some strains were not obtained despite repeated attempts when performing VNTR typing, the number of strains analyzed simultaneously by spoligotyping and VNTR methods was 1053. The ratio of the Beijing genotype obtained for Beijing was still 83.8% (n = 1053) (Table 5) very similar to 82% (n = 1585). These results indicate that our data does represent the TB situation in Beijing.

We believe that choice of appropriate VNTR loci in this study was critical for identifying the prevalent cluster in Beijing. In order to facilitate efficient genotyping of large numbers of strains from Beijing, we first selected a limited panel of typical VNTR loci that would correctly cluster the bacteria into the major clades and genotypic families. 15-locus and 24-locus VNTR genotype methods were commonly being considered to be optimized and were regarded as classical methods [10]. However, recent studies have shown that these loci were not always suited to studies of East Asian strains, especially in China where there is both a high incidence of TB and a high prevalence of the Beijing family strain [30]. Many studies have shown that the discriminatory power of the 15-locus VNTR method was low when applied to the analysis of strains collected from the Chinese mainland [31], and 24-locus VNTR methods are too complex when used for the analyze multiple strains acquired from various regions of the Chinese mainland [32].

The same situation has been encountered in Japan, which also has a high prevalence of Beijing family strains. Studies there have suggested that 15 and 24-locus VNTR methods were poorly optimized for genotyping strains encountered in Japan. In 2008, Murase et al. [15] optimized JATA-12 VNTR loci for the analysis of Japanese strains. They found that these loci had the same discriminatory power as the 24-locus VNTR and were better adapted to East Asian countries which possess a high percentage of Beijing family strains. Gao et al. [16] described several methods in 2006. Their purpose was to identify the simplest and most accurate typing methods, and they found that the 16-locus VNTR method was the most suitable, especially for Beijing family strains. Recently, VNTR-15 and 8 single nucleotide polymorphisms (SNPs) methods have been standardized in Shanghai for the genotyping of Beijing strains [31]. Additional typing of three hypervariable loci (VNTR3820, VNTR4120, and VNTR3232) has also been used; Gao and colleagues have recommended combining the standard VNTR-15 and SNPs as first-line typing methods, and using hypervariable loci for second-line typing of clustered strains in molecular epidemiology studies of homogenous TB populations. However, the above reports have the same shortcoming: the number of strains was too small (325 in Japan, 289 in Shanghai), and thus the results were likely not representative of the broader situation; although they may be suitable for Japan or Shanghai, they may not be appropriate for Beijing. Our purpose here was to identify the most appropriate loci specific to the Beijing metropolitan area. The discriminative power of some loci was too low when apply to Beijing genotype strains. Other loci were excluded because PCR products of these loci were not consistently obtained despite repeated attempts. Other more loci needed more an in-depth study to determine when applies to Beijing genotype strains in different areas.

Which loci were the most suitable for analyzing Beijing district strains? Recently, precise answers are still not been known. If the discriminatory power of loci is too low, unrelated strains will be clustered together. However, if the discriminatory power of loci is too high, related strains will not be clustered. Our study was motivated by the need to select appropriate loci which not only discern the diversity of unrelated strains but also connect stains that can be clustered. Therefore, in this study, we identified 12 VNTR loci, QUB-11b, MIRU10, Mtub21, MIRU23, MIRU39, MIRU16, MIRU40, MIRU31, Mtub24, and Mtub04, MIRU20, and QUB-4156c, as useful genotyping loci; these loci were selected to describe the polymorphisms of these strains. Our newly established 12-locus VNTR typing method had almost equivalent discriminatory power for TB genotyping to that of the 24-locus VNTR (Table 8), especially when applied to Beijing family strains, even though it only contained 12 loci. Our results indicate that this 12-locus (BJ) VNTR method gives appropriate results and HGDI discriminatory power. Thus, this method may be a reference for other typing methods and genotype studies.

The results obtained on the basis of 12-locus (BJ) VNTR typing were slightly different with those of spoligotyping, but this may be due to the presence of two different isolates in some samples. We acquired the cluster result from the minimum spanning tree of spoligotype and VNTR data by BioNumerics software. These clustered strains by VNTR strongly suggest the possibility of the recent TB transmission, which need we investigate the background information of these patients carried clustered strains in the following study.

Our results present a comprehensive picture of the prevalence of different TB genotypes in Beijing and their distribution. Although the major epidemiological cluster identified was the Beijing genotype, considerable genetic diversity was present among the TB strains collected in Beijing. The Beijing family, which is distributed extensively around the world, represents a high proportion of TB strains responsible for ongoing transmission of TB in China, Japan, Korea, Russia, and Central Asia [33]–[35]. This work represents an important study on TB in the Beijing metropolitan area, and our hope is that it will facilitate new government policies and methods for controlling TB, especially in Beijing city.

To the best of our knowledge, this was the first extended and detailed genotyping study of the genetic diversity of TB in a metropolitan area of China using classical two genotyping approaches. This study has important public health implications for Beijing as well as other highly-populated regions of the world. We describe not only the genotypic structure of TB, including the presence of different genotype families dispersed in different districts of the city. Genotyping data can provide a broader understanding of TB epidemiology and outbreaks, information which can be applied to future TB control efforts. This study will add to the reference database of TB strains circulating in Beijing, and may facilitate the implementation of a more efficient anti-TB control program.

## Materials and Methods

### Collection and identification of clinical strains isolates from Beijing

A total of 1585 clinical strains, collected in Beijing, China, during 2009, were obtained from the Beijing Research Institute for Tuberculosis Control. All *M. tuberculosis* strains isolates were from new pulmonary TB patients, who were sputum smear-positive and/or culture-positive for *M. tuberculosis*. The isolates were recovered from −80°C stocks and were subcultured on solid Lowenstein–Jensen medium for 3–4 weeks at 37°C. The 1585 isolates were first assessed by spoligotyping, and then 1056 of these isolates were used to further optimize the highly polymorphic VNTR typing method by set to calculating the Hunter-Gaston discriminatory index (HGDI) values of the VNTR loci.

### Ethics statement

These patients were able to access the study after they signed an Informed Consent form. The protocols applied in this study were approved by the Ethics Committee of the Beijing Research Institute for Tuberculosis Control. The retrospective study was approved by the Institutional Review Boards of Beijing Tuberculosis & Thoracic Tumor Research Institute.

### Spoligotyping

Spoligotyping was performed according to a standard protocol [36]. Spoligotyping was used to identify the genotype of TB strain in the direct repeat (DR) locus as described previously [37]. Genomic DNA was extracted from freshly cultured bacteria. Bacteria were resuspended in 400 µL TE buffer (pH 8. 0), then heated in 100 degrees Celsius bath for 30 min. After incubating on ice for 2 min, samples were centrifuged at 10,000 g for 2 min. Genomic DNA in the supernatant was used as a PCR template. A commercially available Isogen Spoligotyping kit (Isogen Bioscience BV, the Netherlands) was used according to the manufacturer’s instructions. All genomic DNA was amplified with primers DRa (5′-GGTTTTGGGTCTGACGAC-3′) (biotinylated 5′ end) and DRb (5′-CCGAGAGGGGACGGAAAC-3′). Amplified products were then hybridized with a membrane. Images were detected with a chemiluminescence system, including the ECL detection liquid (GE Healthcare, Life Sciences) and X-ray films (Kodak, Rochester, NY). Beijing genotype strains were defined as those which hybridized to all of the last nine spacer oligonucleotides (spacers 35 to 43), while Beijing-like genotype strains were ones that hybridized to only some of the last nine spacers. The results were compared to the SpolDB4 database (an international spoligotype database at the Institute Pasteur de Guadeloupe. http://www.pasteur-guadeloupe.fr:8081/SITVITDemo/) [7].

### VNTR typing

Sequences of primers used for amplification of the nine MIRU loci (MIRU-10, 16, 20,23,24,27,31,39 and 40), two QUB loci (QUB-11b, QUB-4156C) and one Mtub locus (Mtub21) selected and applied to these strains isolates collected in 2009 were listed in Table 4.

For each of the VNTR loci, we used a total PCR reaction volume of 20 µL. Each PCR reaction contained the following: 1 µL of DNA template; a 0.4 µM of each primer (Sbs Biotech, Beijing, China), 2×Taq PCR Master Mix (Tiangen Biotech, Beijing, China), and double-distilled H_2_O to bring the total volume to 20 µL. Then PCR reaction conditions were as follows: initial denaturation at 95°C for 5 min, and then 35 cycles of denaturation at 95°C, annealing for 30 s at a temperature range 58–62°C and extension at 72°C for 45 s, followed by a final extension at 72°C for 7 min. PCR products were analyzed by electrophoresis in 2% agarose gels, using a 50 bp DNA ladder (TaKaRa-Bio Inc., Dalian, China) as the reference marker. H37Rv and double-distilled H_2_O were used as positive and negative controls, respectively. The copy number of repeats was calculated using the following formula: (length of the PCR product minus length of the flanking regions)/length of one repeat copy unit.

### Data analysis

Spoligotyping data, expressed in binary and octal formats, and VNTR data expressed in decimal format were analyzed using BioNumerics software (Version 5.0, Applied Maths, Belgium). Cluster analysis was performed and a dendrogram was generated in Bionumerics using the Dice similarity coefficient and UPGMA coefficient. Genotype analyses were performed in NatureEdge software (own).

### Statistical analysis

The discriminatory power (the Hunter-Gaston discriminatory index (HGDI)) of each typing method was calculated according to a previously published method [38]:
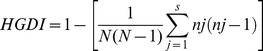
where *N* is the total number of isolates in the typing method, *s* is the number of distinct patterns discriminated by VNTR, and 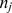 is the number of isolates belonging to the *j*th pattern. The percentage clustering was calculated with the following formula: 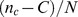, where *N* is the total number of isolates, *C* is the number of clusters, and *n_c_* is the total number of clustered isolates [39].

## Supporting Information

Figure S1 **Map of Beijing showing the distribution of strains collected in various districts from Beijing.**
(TIF)

Figure S2 **The VNTR cluster of isolate strains collected in various districts from Beijing 2009 by BioNumerics.**
(PDF)

Table S1 **Description of most popular shared-types (SITs; n = 1565 isolates) and new found orphan isolates with corresponding spoligotyping defined lineagessublineages from M.tuberculosis.**
(DOC)

Table S2 **The comparison between Beijing family genotype and Non-Beijing family genotype in sex, age, and household by Spoligotyping result (n = 1585).** There was a statistically significant difference in sex, but not found statistically significant associations with age and household registration.
(DOCX)

Table S3 **The clad structure of M. tuberculosis isolates strains from Beijing in 2009 year.** The population clad structure mainly based on major M. tuberculosis complex clades.
(DOC)

Table S4 **Spoligotype and VNTR original data of isolates strains from Beijing in 2009 year.** The spoligotyping profiles of the 1565 isolates, and the 12-locus(BJ) VNTR repeats profiles of the 1053 isolates.
(XLS)
