# Supplementary figures and images for: Genotypic Diversity Analysis of Mycobacterium tuberculosis Strains Collected from Beijing in 2009, Using Spoligotyping and VNTR Typing
Source: PLoS One. 2014 Sep 19;9(9):e106787. doi: 10.1371/journal.pone.0106787 (PMC4169523; doi:10.1371/journal.pone.0106787)

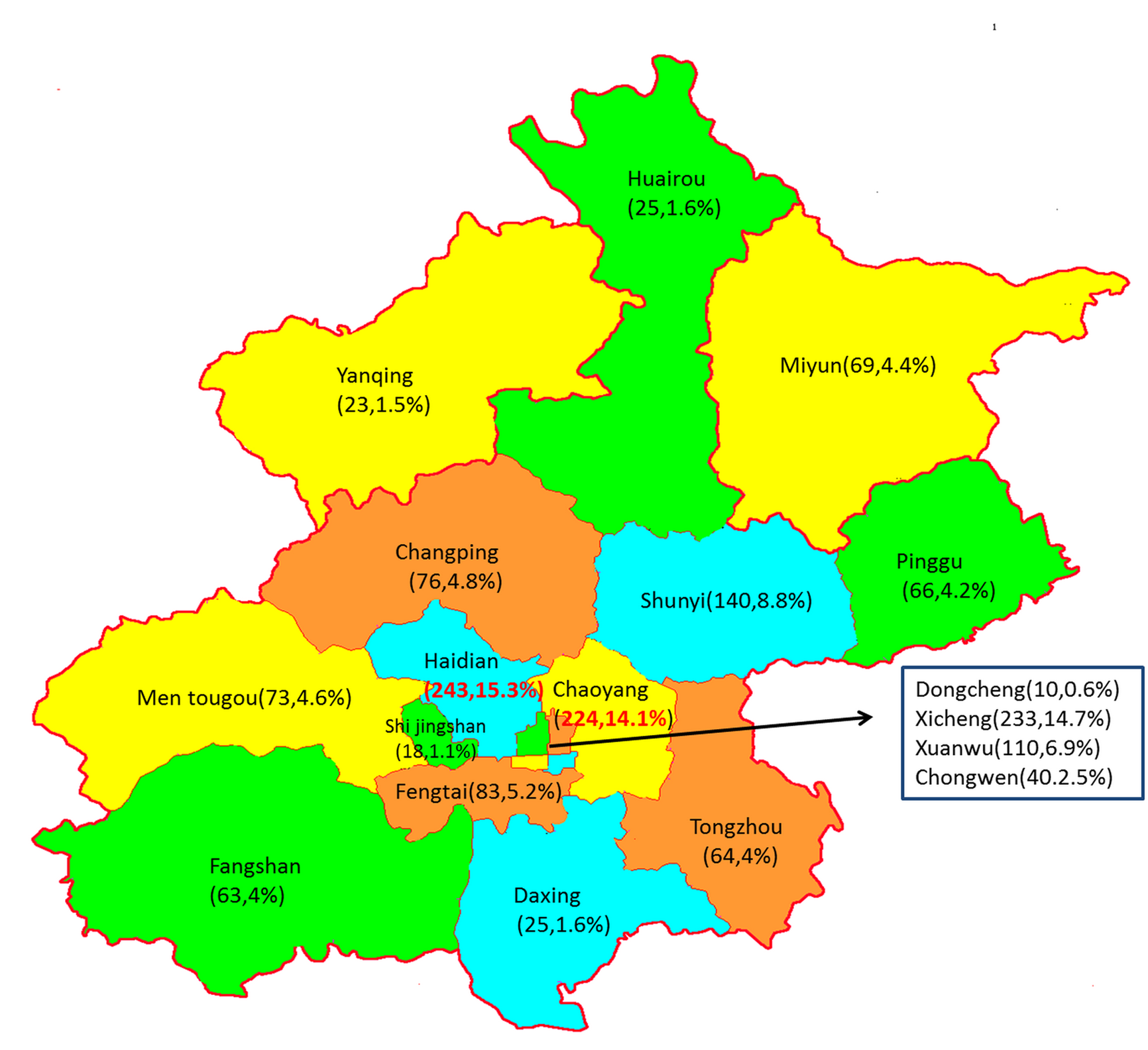

Supplement: Figure S1 — Map of Beijing showing the distribution of strains collected in various districts from Beijing. (TIF) [file pone.0106787.s001.tif]
