# Supplementary material for: Genotypic Diversity Analysis of Mycobacterium tuberculosis Strains Collected from Beijing in 2009, Using Spoligotyping and VNTR Typing
Source: PLoS One. 2014 Sep 19;9(9):e106787. doi: 10.1371/journal.pone.0106787 (PMC4169523; doi:10.1371/journal.pone.0106787)
Supplement: Table S1 — Description of most popular shared-types (SITs; n = 1565 isolates) and new found orphan isolates with corresponding spoligotyping defined lineagessublineages from M.tuberculosis. (DOC) [file pone.0106787.s003.doc]

Table S1. Description of most popular shared-types (SITs; n = 1565 isolates) and Unknown new isolates strains with corresponding spoligotyping defined lineages/sublineages from *M.tuberculosis* isolates of Beijing in 2009.

| SIT* | Spoligotype description | Octal number | Number (%)  in study^$^ | Family^#^ | Clustered  vs. unique  patterns |
| --- | --- | --- | --- | --- | --- |
| 1  7  37  40  50  51  52  53  54  127  131  167  175  190  255  260  265  269  278  334  357  462  482  505 | 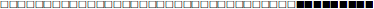      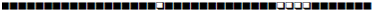                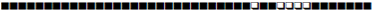    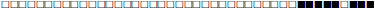  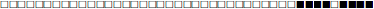  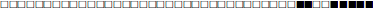  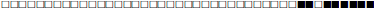  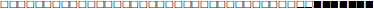      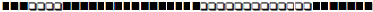  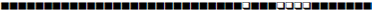  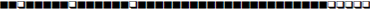 | 000000000003771  377777777760771  777777777760700  777777377760771  777777777720771  777777777760700  777777777760731  777777777760771  777777777763771  000000000003731  777777760760731  777777777660771  777777677760731  777717777760771  000000000003671  000000000003171  000000000003371  000000000000771  777777777760761  577777777760771  703777740000771  777777777560771  676773777777600  777737777760700 | 1224(77.18%)  1  6(0.37%)  2(0.12%)  2(0.12%)  1  18(1.13%)  45(2.83%)  67(4.22%)  1  2(0.12%)  1  1  18(1.13%)  2(0.13%)  2(0.13%)  1  10(0.63%)  1  2(0.12%)  1  1  2(0.12%)  1 | Beijing  T1  T3  T4  H3  T1  T2  T1  MANU2  H4  T1  T1  T2  Beijing like  Beijing like  Beijing like  Beijing like  Beijing like  T1  T1  CAS1-Delhi  T1  BCG  T3 | Clustered  Unique  Clustered  Clustered  Clustered  Unique  Clustered  Clustered  Clustered  Clustered  Unique  Clustered  Unique  Unique  Clustered  Clustered  Unique  Unique  Clustered  Clustered  Unique  Unique  Clustered  Unique |

| SIT | Spoligotype description | Octal number | Number (%)  in study | Lineage | Clustered  vs. unique  patterns |
| --- | --- | --- | --- | --- | --- |
| 515  520  522  523  535  541  574  583  612  621  632  742  787  796  803  875  895  926  941  966  1088  1162  1163  1166  1168 | 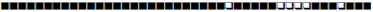        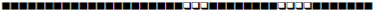  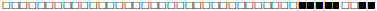          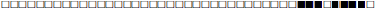      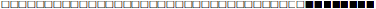          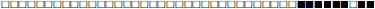            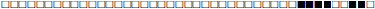 | 777777776760731  777777777760571  777777777760770  777777777777771  777777707760771  000000000003711  777777777740071  777737777763771  777777777760751  000000000002771  000000000003571  777777770020771  777777777760071  000000000001771  777740007760771  777717777760731  677777477013771  773777777760771  000000000003751  775777777760771  777767777763771  000000000002171  677737777760771  777377777760771  000000000003631 | 2(0.12%)  2(0.12%)  1  8(0.50%)  1  1  1  3(0.18%)  1  3  3(0.18%)  1  1  1  3(0. 18%)  1  1  1  8(0.51%)  2(0.12%)  2(0.12%)  1  1  1  1 | T2  T1  T1  U  T1  Beijing like  U  MANU2  T1  Beijing like  Beijing like  H3  U  Beijing like T1  T2  T3  T1  Beijing like  T1  MANU2  Beijing like  T3  T1  Beijing like | Clustered  Clustered  Unique  Clustered  Unique  Unique  Unique  Clustered  Unique  Clustered  Clustered  Unique  Clustered  Clustered Clustered Clustered Unique  Clustered  Unique Clustered  Unique Unique Unique  Unique  Clustered |

| SIT | Spoligotype description | Octal number | Number (%)  in study | Lineage | Clustered  vs. unique  patterns |
| --- | --- | --- | --- | --- | --- |
| 1192  1211  1247  1302  1364  1580  1626  1674  1688  1793  New  New  New  New  New  New  New  New New  New  New  New | 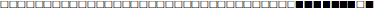      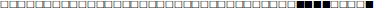  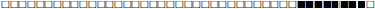  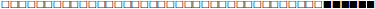  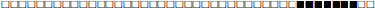  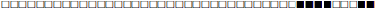  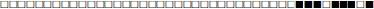  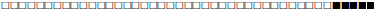  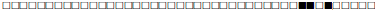  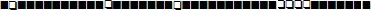  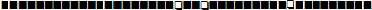  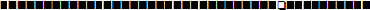  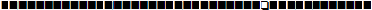 | 777777677763771  576377777760771  777777607763771  577777777760731  000000000003471  777777747760771  777777776760771  000000000003761  777777777777771  777777777760000  000000000003601  000000000003770  000000000000371  000000000003760  000000000003611  000000000003561  000000000000171  000000000003200  577737677760771  777777667773771  777777777767771  777777777737771 | 3(0.18%)  1  1  2(0.12%)  1  1  1  2(0.12%)  3(0.18%)  1  1  1  1  2(0.12%)  2(0.12%)  1  3(0.19%)  1  2(0.12%)  2(0.12%)  3(0.18%)  2(0.12%) | MANU2  S  MANU2  T2  Beijing like  T1  T1  Beijing like  T1  U  Beijing like  Beijing like  Beijing like  Beijing like  Beijing like  Beijing like  Beijing like  Beijing like  Unknown  Unknown  Unknown  Unknown | Unique  Unique Clustered  Clustered  Unique  Clustered  Clustered  Unique  Unique  Unique  Unique  Clustered  Unique  Clustered  Clustered  Unique  Clustered  Unique  Clustered  Clustered  Clustered  Clustered |

| SIT | Spoligotype description | Octal number | Number (%)  in study | Lineage | Clustered  vs. unique  patterns |
| --- | --- | --- | --- | --- | --- |
| New New  New  New New  New  New  New  New  New  New  New  New  New  New  New  New  New  New  New  New  New  New  New | 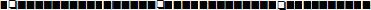  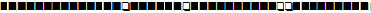  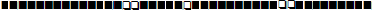  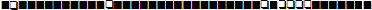  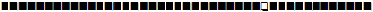  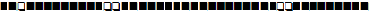  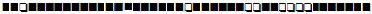  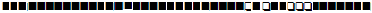  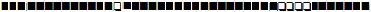  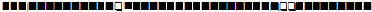  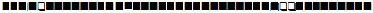  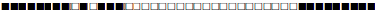  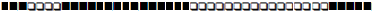  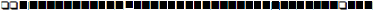  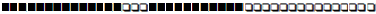  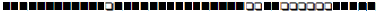  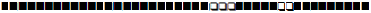  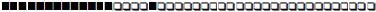  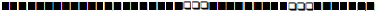  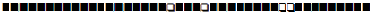  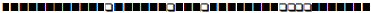  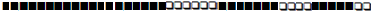  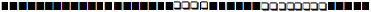  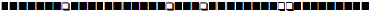 | 577777377767771  777757677763771  777747677743771  577737777720771  777777777737771  777717777763771  677777777460771  777777777530771  777757777760731  777757777763771  757777777763771  776560000003771  703777700000171  177777777777731  777743777400000  777737777760171  777777770763771  777741000000000  777777707770771  777777567763771  777737777760771  777777403760760  777777607700171  775777777763771 | 2(0.12%)  2(0.12%)  3(0.18%)  1  2(0.12%)  1  1  1  1  2(0.12%)  2(0.12%)  3(0.18%)  1  1  1  1  1  1  1  1  1  1  1  1 | Unknown  Unknown  Unknown  Unknown Unknown  Unknown  Unknown  Unknown  Unknown  Unknown  Unknown  Unknown  Unknown  Unknown  Unknown  Unknown  Unknown  Unknown  Unknown  Unknown  Unknown  Unknown  Unknown  Unknown | Clustered  Clustered Clustered Unique  Clustered  Unique  Unique  Unique  Unique  Clustered  Clustered  Clustered  Unique  Unique  Unique  Unique  Unique  Unique  Unique  Unique  Unique  Unique  Unique  Unique |

| SIT | Spoligotype description | Octal number | Number (%)  in study | Lineage | Clustered  vs. unique  patterns |
| --- | --- | --- | --- | --- | --- |
| New New  New  New New  New  New  New  New  New  New  New  New  New  New  New  New  New  New  New  New  New | 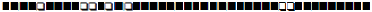  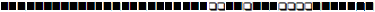  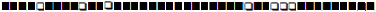  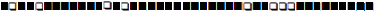  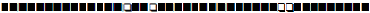  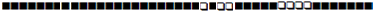  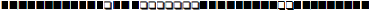  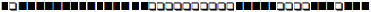  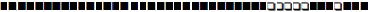  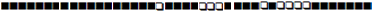  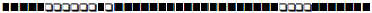  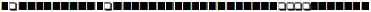  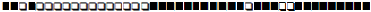  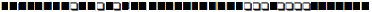  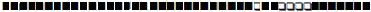  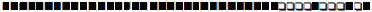  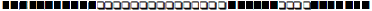  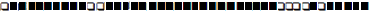  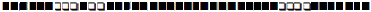  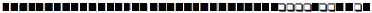  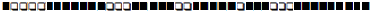  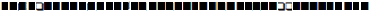 | 757127777763771  777777771560771  757337777543771  557727777543771  777767677763771  777777764760771  777734017763771  577776000760731  777777777740731  777717361720771  760177777760771  577737777760771  600003777563771  776657777420771  777777777660071  777777777760421  777600001760771  377477777760571  770477777760771  777777777760461  417707637743771  757777777763771 | 1  1  1  1  1  1  1  1  1  1  1  1  1  1  1  1  1  1  1  1  1  1 | Unknown  Unknown  Unknown  Unknown Unknown  Unknown  Unknown  Unknown  Unknown  Unknown  Unknown  Unknown  Unknown  Unknown  Unknown  Unknown  Unknown  Unknown  Unknown  Unknown  Unknown  Unknown | Unique  Unique  Unique  Unique  Unique  Unique  Unique  Unique  Unique  Unique  Unique  Unique  Unique  Unique  Unique  Unique  Unique  Unique  Unique  Unique  Unique  Unique |

| SIT | Spoligotype description | Octal number | Number (%)  in study | Lineage | Clustered  vs. unique  patterns |
| --- | --- | --- | --- | --- | --- |
| New New  New  New New  New  New  New  New  New  New | 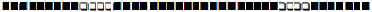  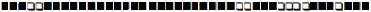  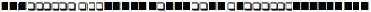  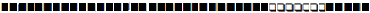  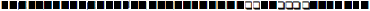  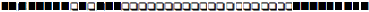  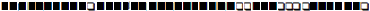  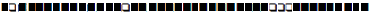  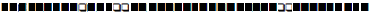  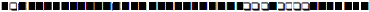  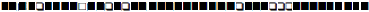 | 777003777760771  717777777160731  700077346403771  777777777740171  677777777460771  776560000003771  777577777760770  577767777743771  777347777763771  577777777420771  757327777543771 | 1  1  1  1  1  1  1  1  1  1  1 | Unknown  Unknown  Unknown  Unknown Unknown  Unknown  Unknown  Unknown  Unknown  Unknown  Unknown | Unique  Unique  Unique  Unique  Unique  Unique  Unique  Unique  Unique  Unique  Unique |

* SIT number from SpoIDB4.0; SIT, spoligotype international type.

# Spoligotype families as assigned in the Spoldb4.0 database.

$ Number of isolates with a common SIT and the percentage of strains with a common SIT among all strains in this study.
