# Supplementary material for: Genotypic Diversity Analysis of Mycobacterium tuberculosis Strains Collected from Beijing in 2009, Using Spoligotyping and VNTR Typing
Source: PLoS One. 2014 Sep 19;9(9):e106787. doi: 10.1371/journal.pone.0106787 (PMC4169523; doi:10.1371/journal.pone.0106787)
Supplement: Table S2 — The comparison between Beijing family genotype and Non-Beijing family genotype in sex, age, and household by Spoligotyping result (n = 1585). There was a statistically significant difference in sex, but not found statistically significant associations with age and household registration. (DOCX) [file pone.0106787.s004.docx]

Table S2. The comparison between Beijing family genotype and non-Beijing family genotype in sex, age, and household by Spoligotyping result (n=1585).

| Variable | Beijing family genotype | non-Beijing family genotype | OR | 95%CI | P  Value |
| --- | --- | --- | --- | --- | --- |
| Total patients  sex  Male  Female  Age  ≤45  >45  House hold  City  Other | 1300  907  393  870  430  626  674 | 285  233  52  180  105  154  131 | 0.515  1.180  0.790 | 0.373-0.711 | 0.001 |
|  |  |  |  |  |  |
|  |  |  |  | 0.904-1.541 | 0.223 |
|  |  |  |  | 0.611-1.022 | 0.072 |
|  |  |  |  |  |  |

OR: odd ratio

CI: confidence interval.
