# Supplementary material for: Genotypic Diversity Analysis of Mycobacterium tuberculosis Strains Collected from Beijing in 2009, Using Spoligotyping and VNTR Typing
Source: PLoS One. 2014 Sep 19;9(9):e106787. doi: 10.1371/journal.pone.0106787 (PMC4169523; doi:10.1371/journal.pone.0106787)
Supplement: Table S3 — The clad structure of M. tuberculosis isolates strains from Beijing in 2009 year. The population clad structure mainly based on major M. tuberculosis complex clades. (DOC) [file pone.0106787.s005.doc]

Table S3. The clad structure of *M. tuberculosis* isolates strains from Beijing in 2009.

| Year | East Asian (Beijing) (%) | Euro-American (%) | Indo-Oceanic (%) | Mycobacterium bovis (%) | Unknown (%) | Total (n) |
| --- | --- | --- | --- | --- | --- | --- |
| 2009  Total(%) | 1300(82.02)  1300(82.02) | 151(9.53)  151(9.53) | 131(8.26)  131(8.26) | 2(0.13)  2(0.13) | 1(0.06)  1(0.06) | 1585  1585 |
