# Supplementary material for: Genotypic Diversity Analysis of Mycobacterium tuberculosis Strains Collected from Beijing in 2009, Using Spoligotyping and VNTR Typing
Source: PLoS One. 2014 Sep 19;9(9):e106787. doi: 10.1371/journal.pone.0106787 (PMC4169523; doi:10.1371/journal.pone.0106787)

Categorical
VNTR

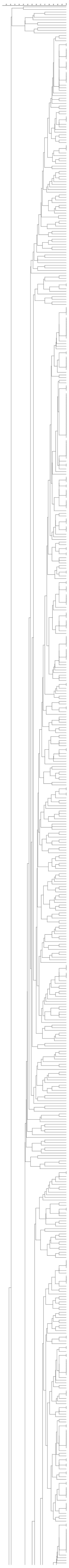

VNTR

Mtub21	QUB-11b	QUB-4156c	MIRU10	MIRU16	MIRU31	MIRU39	MIRU40	ETR A	Mtub30	Mtub39	MIRU23	Key
2	3	2	2	3	5	7	6	3	4	4	3	BJTB090261
2	3	2	5	5	5	5	2	0	4	4	5	BJTB091346
3	1	2	6	5	4	5	2	4	4	4	5	BJTB091220
3	1	3	6	5	4	4	2	3	4	4	5	BJTB091388
3	1	2	3	5	6	5	2	3	4	3	5	BJTB091440
3	1	2	6	5	5	4	4	3	4	5	5	BJTB091387
3	1	5	5	3	4	3	2	4	4	4	5	BJTB090035
3	1	3	5	4	5	3	2	3	4	4	5	BJTB091930
5	1	3	3	6	3	2	2	2	4	4	5	BJTB090562
5	1	2	3	5	5	4	2	2	4	4	5	BJTB091871
3	1	3	3	3	3	4	2	3	4	4	5	BJTB091788
5	6	2	4	4	4	9	3	4	4	4	5	BJTB090879
5	6	2	4	4	4	4	3	4	4	4	5	BJTB091014
5	6	2	4	4	6	9	3	4	4	4	5	BJTB090890
5	6	2	4	4	5	5	3	4	4	4	5	BJTB091033
5	6	2	4	4	5	5	3	4	4	4	5	BJTB091036
5	6	2	4	4	5	4	3	4	4	4	5	BJTB090982
5	6	2	4	4	5	4	3	4	4	4	5	BJTB090985
5	6	2	4	4	5	4	3	4	4	4	5	BJTB090990
5	6	2	4	4	5	4	3	4	4	4	5	BJTB091018
5	6	2	4	4	5	4	3	4	4	4	5	BJTB091888
5	6	2	4	4	5	3	3	4	4	4	5	BJTB090867
5	6	2	4	4	5	3	3	4	4	4	5	BJTB090997
5	6	2	4	4	5	3	3	4	4	4	5	BJTB091001
5	6	2	4	4	5	3	3	4	4	4	5	BJTB091029
5	6	2	4	4	5	9	3	4	4	4	5	BJTB090861
5	6	2	4	4	5	9	3	4	4	4	5	BJTB090869
5	6	2	4	4	5	9	3	4	4	4	5	BJTB090901
5	6	2	4	4	5	9	3	4	4	4	5	BJTB090914
5	6	2	4	4	5	9	3	4	4	4	5	BJTB090917
5	6	2	4	4	5	8	3	4	4	4	5	BJTB090862
5	6	2	4	4	5	7	3	4	4	4	5	BJTB090882
5	6	2	4	4	5	2	3	4	4	4	5	BJTB091005
5	6	2	4	4	3	3	3	4	4	4	5	BJTB091000
5	3	2	4	4	5	4	3	4	4	4	5	BJTB090964
5	6	2	5	4	5	3	3	4	4	4	5	BJTB090745
5	6	2	3	4	5	3	3	4	4	4	5	BJTB091017
5	6	2	4	4	5	3	3	4	4	3	5	BJTB091008
5	6	2	4	6	5	4	3	4	4	4	5	BJTB090986
5	6	2	4	6	5	6	3	4	4	4	5	BJTB091205
9	5	2	4	4	5	9	3	4	4	4	5	BJTB090884
4	5	2	4	4	5	9	3	4	4	4	5	BJTB090893
5	5	2	4	4	5	9	3	4	4	4	5	BJTB090887
5	5	2	4	4	5	9	3	4	4	4	5	BJTB090898
5	5	2	4	4	5	9	3	4	4	4	5	BJTB090908
5	5	2	4	4	5	3	3	4	4	4	5	BJTB091004
5	5	2	4	4	5	4	3	4	4	4	5	BJTB090989
5	5	2	4	4	5	9	3	4	5	4	5	BJTB090870
5	2	2	4	4	5	9	3	4	4	4	5	BJTB090906
5	4	2	4	4	5	2	3	4	4	4	5	BJTB090910
5	4	2	4	4	5	3	3	4	4	4	5	BJTB090998
4	4	2	4	4	5	9	3	4	4	4	5	BJTB090875
5	4	2	4	4	5	9	3	4	4	4	5	BJTB090918
4	6	2	4	4	5	4	3	4	4	4	5	BJTB090981
4	6	2	4	4	5	4	3	4	4	4	5	BJTB091011
4	6	2	4	4	5	4	3	4	4	4	5	BJTB091022
4	6	2	4	4	5	9	3	4	4	4	5	BJTB090871
4	6	2	4	4	5	9	3	4	2	4	5	BJTB090913
4	7	2	4	4	5	9	3	4	4	4	5	BJTB090883
4	7	2	4	4	5	2	3	4	4	4	5	BJTB091020
4	20	2	4	4	5	9	3	4	4	4	5	BJTB090895
5	5	2	4	4	5	3	3	4	4	2	5	BJTB090992
5	5	2	4	4	5	3	3	4	3	4	5	BJTB091007
5	6	2	4	4	5	9	3	3	4	4	5	BJTB090921
5	6	2	4	4	5	4	3	3	4	4	5	BJTB090991
5	6	2	4	4	5	3	3	3	4	4	5	BJTB090868
5	5	2	4	4	5	3	3	3	4	4	5	BJTB091025
5	5	2	4	4	5	5	3	3	4	4	5	BJTB091032
5	3	2	4	4	5	2	3	3	4	4	5	BJTB091013
5	5	4	4	4	5	3	3	3	4	4	5	BJTB091021
6	6	2	4	4	5	5	3	3	4	4	5	BJTB091034
5	5	2	4	4	3	4	3	3	4	4	5	BJTB090983
5	5	4	4	4	5	2	3	4	4	4	5	BJTB090894
5	5	4	4	4	5	9	3	4	4	4	5	BJTB090912
5	6	4	4	4	5	9	3	4	4	4	5	BJTB090904
5	5	4	4	4	5	2	5	4	4	4	5	BJTB091023
5	6	3	4	4	5	2	3	4	4	4	5	BJTB090892
5	5	3	4	4	5	2	3	4	4	4	5	BJTB091002
5	5	3	4	4	5	5	3	4	4	4	5	BJTB091035
5	5	3	4	4	4	2	3	4	4	4	5	BJTB090896
5	5		4	4	5	9	3	3	4	6	5	BJTB090865
5	5	2	4	4	5	9	3	4	4	6	5	BJTB090889
5	6	2	3	4	5	9	3	4	4	4	5	BJTB090915
5	6	2	3	4	5	9	3	4	4	5	5	BJTB090924
5	5	2	3	4	5	9	3	4	4	4	5	BJTB090919
5	6	2	3	6	5	9	3	4	4	4	0	BJTB090925
6	5	3	4	4	5	9	3	4	4	4	5	BJTB090866
3	6	3	4	4	5	9	3	4	4	4	5	BJTB090900
8	6	3	4	4	5	9	3	4	4	7	5	BJTB090874
5	5	2	4	4	5	9	4	3	4	4	5	BJTB090877
5	5	2	4	4	3	9	4	4	4	4	5	BJTB090880
5	5	2	4	4	4	2	4	4	4	4	5	BJTB090909
5	4	2	4	4	4	9	4	4	4	4	5	BJTB090878
4	4	2	4	4	4	9	3	4	4	4	5	BJTB090911
5	7	4	4	4	5	9	2	4	4	4	5	BJTB090876
4	4	2	3	4	5	2	3	4	4	4	5	BJTB090567
4	4	2	4	4	5	3	3	4	2	4	5	BJTB090993
4	7	2	4	4	2	4	3	4	2	4	5	BJTB090971
4	3	2	4	4	5	4	2	4	4	4	5	BJTB090965
6	5	2	3	4	6	4	3	4	4	4	5	BJTB090134
6	7	2	4	4	4	4	3	4	4	4	5	BJTB090872
3	6	2	4	4	4	4	3	4	4	6	5	BJTB090968
4	5	3	4	4	5	5	3	4	3	4	5	BJTB091028
4	7	2	4	6	8	5	3	4	4	4	5	BJTB091367
4	7	2	4	6	6	5	3	4	4	4	5	BJTB091525
4	7	2	4	5	5	8	3	4	4	4	5	BJTB090903
4	7	2	4	7	7	6	3	4	4	4	5	BJTB091447
4	7	2	4	7	7	6	3	4	4	4	5	BJTB091467
4	7	2	4	7	6	6	3	4	4	4	5	BJTB091299
4	7	2	4	7	7	3	3	4	4	5	5	BJTB091558
4	10	2	2	7	5	6	3	4	4	4	5	BJTB091394
4	2	2	6	7	6	5	3	4	2	4	5	BJTB091399
4	7	2	4	7	6	5	3	4	2	4	5	BJTB091680
4	6	2	4	7	6	5	3	4	3	4	5	BJTB091289
8	4	2	4	7	6	5	3	4	3	4	5	BJTB091684
5	6	2	4	7	6	4	3	4	4	4	5	BJTB091524
5	6	2	4	7	6	4	3	4	4	4	5	BJTB091633
5	6	2	4	7	6	4	3	4	4	4	5	BJTB091635
5	6	2	4	7	6	4	3	4	4	4	5	BJTB091636
5	6	2	4	7	6	5	3	4	4	4	5	BJTB091359
5	6	2	4	7	6	5	3	4	4	4	5	BJTB091375
5	6	2	4	7	6	5	3	4	4	4	5	BJTB091385
5	6	2	4	7	6	5	3	4	4	4	5	BJTB091411
5	6	2	4	7	6	5	3	4	4	4	5	BJTB091534
5	6	2	4	7	6	5	3	4	4	4	5	BJTB091536
5	6	2	4	7	6	5	3	4	4	4	5	BJTB091539
5	6	2	4	7	6	5	3	4	4	4	5	BJTB091540
5	6	2	4	7	6	5	3	4	4	4	5	BJTB091563
5	6	2	4	7	6	3	3	4	4	4	5	BJTB091656
5	6	2	5	7	6	5	3	4	4	4	5	BJTB091548
5	6	2	4	7	6	5	7	4	4	4	5	BJTB091335
5	6	0	4	7	6	5	3	4	4	4	5	BJTB091428
5	5	2	4	7	6	5	3	3	4	4	5	BJTB091554
5	5	2	4	7	6	5	3	3	4	4	5	BJTB091576
5	5	2	4	7	6	5	3	4	4	4	5	BJTB091379
5	5	2	4	7	6	5	3	4	4	4	5	BJTB091386
5	5	2	4	7	6	5	3	4	4	4	5	BJTB091429
5	5	2	4	7	6	5	3	4	4	4	5	BJTB091526
5	5	2	4	7	6	5	3	4	4	4	5	BJTB091560
5	5	2	4	7	6	5	3	4	4	4	5	BJTB091579
5	7	2	4	7	6	5	3	4	4	4	5	BJTB091569
6	6	2	4	7	6	5	3	4	4	4	5	BJTB091383
6	4	2	4	7	6	5	3	4	4	4	5	BJTB091535
5	6	2	4	7	9	5	3	4	4	4	5	BJTB091373
5	6	2	4	7	4	5	3	4	4	4	5	BJTB091426
5	6	2	3	7	7	5	3	4	4	4	5	BJTB091503
5	6	2	3	7	7	5	3	4	4	4	5	BJTB091570
5	6	2	3	7	7	5	3	4	4	4	5	BJTB091689
5	6	2	4	7	7	5	3	4	4	4	5	BJTB091402
5	6	2	4	7	7	5	3	4	4	4	5	BJTB091415
5	6	2	4	7	7	5	3	4	4	4	5	BJTB091491
5	6	2	4	7	7	5	3	4	4	4	5	BJTB091494
5	6	2	4	7	7	5	3	4	4	4	5	BJTB091504
5	6	2	4	7	7	5	3	4	4	4	5	BJTB091505
5	6	2	4	7	7	5	3	4	4	4	5	BJTB091519
5	6	2	4	7	7	5	3	4	4	4	5	BJTB091521
5	6	2	4	7	7	5	3	4	4	4	5	BJTB091523
5	6	2	4	7	7	5	3	4	4	4	5	BJTB091533
5	6	2	4	7	7	5	3	4	4	4	5	BJTB091543
5	6	2	4	7	7	5	3	4	4	4	5	BJTB091547
5	6	2	4	7	7	5	3	4	4	4	5	BJTB091566
5	6	2	4	7	7	5	3	4	4	4	5	BJTB091568
5	6	2	4	7	7	5	3	4	4	4	5	BJTB091572
5	6	2	4	7	7	5	3	4	4	4	5	BJTB091691
5	6	2	4	7	7	5	3	4	4	4	5	BJTB091692
5	6	2	4	7	7	5	3	4	4	4	5	BJTB091697
5	5	2	4	7	7	5	3	4	4	4	5	BJTB091495
5	5	2	4	7	7	5	3	4	4	4	5	BJTB091496
5	5	2	4	7	7	5	3	4	4	4	5	BJTB091498
5	5	2	4	7	7	5	3	4	4	4	5	BJTB091502
5	5	2	4	7	7	5	3	4	4	4	5	BJTB091561
5	5	2	4	7	7	5	3	4	4	4	5	BJTB091596
5	5	2	4	7	7	5	3	4	4	4	5	BJTB091690
5	5	2	4	7	7	5	3	4	4	4	5	BJTB091702
5	8	2	4	7	7	5	3	4	4	4	5	BJTB091696
5	4	2	4	7	7	5	3	4	4	4	5	BJTB091404
5	4	2	4	7	7	5	3	4	4	4	5	BJTB091688
5	7	2	4	7	7	5	3	4	4	4	5	BJTB091530
5	6	2	4	7	7	3	3	4	4	4	5	BJTB091592
5	5	2	4	7	7	5	3	4	2	4	5	BJTB091604
5	6	2	4	7	8	5	3	4	4	4	5	BJTB091350
5	6	2	4	7	8	5	3	4	4	4	5	BJTB091362
5	6	2	4	7	8	5	3	4	4	4	5	BJTB091371
5	7	2	4	7	8	5	3	4	4	4	5	BJTB091341
5	7	2	4	7	8	5	3	4	4	4	5	BJTB091368
5	5	2	4	7	8	5	3	4	4	4	5	BJTB091305
5	5	2	4	7	8	5	3	4	4	4	5	BJTB091308
5	5	2	4	7	8	5	3	4	4	4	5	BJTB091331
5	5	2	4	7	8	5	3	4	4	4	5	BJTB091370
5	4	2	4	7	8	5	3	4	4	4	5	BJTB091298
5	4	2	4	7	8	5	3	4	4	4	5	BJTB091320
5	4	2	4	7	8	5	3	4	4	4	5	BJTB091369
5	4	2	4	7	8	5	3	4	4	4	5	BJTB091374
5	4	2	4	7	8	4	3	4	4	4	5	BJTB091351
5	5	2	4	7	5	5	3	5	4	4	5	BJTB091210
5	5	2	4	7	5	5	3	2	4	4	5	BJTB091248
5	5	2	4	7	5	5	3	4	4	4	5	BJTB091279
5	5	2	4	7	5	5	3	4	4	4	5	BJTB091284
5	5	2	4	7	5	5	3	4	4	4	5	BJTB091381
5	6	2	4	7	5	5	3	4	4	4	5	BJTB091230
5	6	2	4	7	5	5	3	4	4	4	5	BJTB091242
5	6	2	4	7	5	5	3	4	4	4	5	BJTB091283
5	6	2	4	7	5	5	3	2	4	4	5	BJTB091218
5	5	4	4	7	5	5	3	4	4	4	5	BJTB091219
5	6	2	4	7	5	5	1	4	4	4	5	BJTB091190
5	4	2	4	7	5	5	2	4	4	4	5	BJTB091193
5	4	2	4	7	5	5	4	4	4	4	5	BJTB091252
5	4	2	4	7	6	5	3	4	4	4	5	BJTB091281
5	4	2	4	7	6	5	3	4	4	4	5	BJTB091483
5	4	2	4	7	5	5	3	4	4	4	5	BJTB091192
5	4	2	4	7	5	5	3	4	4	4	5	BJTB091275
4	4	2	4	7	5	5	3	4	4	4	5	BJTB091277
4	4	2	4	7	4	5	3	4	4	4	5	BJTB091492
4	4	2	4	7	6	5	3	4	4	4	5	BJTB091377
5	6	2	4	7	6	5	3	4	4	5	5	BJTB091376
5	6	2	4	7	7	5	3	4	4	5	5	BJTB091497
5	6	2	4	7	8	5	3	4	4	5	5	BJTB091306
5	6	2	4	7	8	5	3	4	4	5	5	BJTB091330
5	4	2	4	7	8	5	3	4	4	5	5	BJTB091311
5	6	2	4	7	8	5	3	4	4	6	5	BJTB091325
5	8	2	4	7	7	4	3	4	4	4	5	BJTB091591
5	8	2	4	7	7	4	3	4	4	4	5	BJTB091618
5	6	2	4	7	7	4	3	4	4	4	5	BJTB091589
5	6	2	4	7	7	4	3	4	4	4	5	BJTB091626
5	6	2	4	7	7	4	3	4	4	4	5	BJTB091630
5	5	2	4	7	7	4	3	4	4	4	5	BJTB091460
5	5	2	4	7	7	4	3	4	4	4	5	BJTB091580
5	5	2	4	7	7	4	3	4	4	4	5	BJTB091583
5	5	2	4	7	7	4	3	4	4	4	5	BJTB091588
5	5	2	4	7	7	4	3	4	4	4	5	BJTB091615
5	5	2	4	7	7	4	3	4	4	2	5	BJTB091622
5	6	2	4	7	7	4	3	4	4	4	4	BJTB091590
5	6	2	4	7	8	4	3	4	4	4	5	BJTB091313
5	6	2	4	7	8	4	3	4	4	4	5	BJTB091321
5	6	2	4	7	8	4	3	4	4	4	5	BJTB091338
5	6	2	4	7	5	4	3	4	4	4	5	BJTB091264
5	6	2	4	7	5	4	3	4	4	4	5	BJTB091266
5	6	2	4	7	5	4	3	4	4	4	5	BJTB091269
5	6	2	4	7	5	4	3	4	4	4	5	BJTB091272
5	6	2	4	7	4	4	3	4	4	4	5	BJTB091271
5	6	2	4	7	5	4	3	4	4	2	5	BJTB091262
5	5	2	4	7	7	6	3	4	4	4	5	BJTB091442
5	5	2	4	7	7	6	3	4	4	4	5	BJTB091444
5	5	2	4	7	7	6	3	4	4	4	5	BJTB091456
5	5	2	4	7	7	6	3	4	4	4	5	BJTB091461
5	5	2	4	7	7	6	3	4	4	4	5	BJTB091465
5	6	2	4	7	7	6	3	4	4	4	5	BJTB091432
5	6	2	4	7	7	6	3	4	4	4	5	BJTB091433
5	6	2	4	7	7	6	3	4	4	4	5	BJTB091453
5	6	2	4	7	7	6	3	4	4	4	5	BJTB091468
5	6	2	4	7	7	6	3	4	4	4	5	BJTB091477
5	3	2	4	7	7	6	3	4	4	4	5	BJTB091457
5	4	2	4	7	7	6	3	4	4	4	5	BJTB091475
5	5	2	3	7	7	6	3	4	4	4	5	BJTB091473
5	5	0	4	7	7	6	3	4	4	4	5	BJTB091459
6	6	2	4	7	7	6	3	4	4	4	5	BJTB091452
5	4	2	4	7	5	6	3	4	4	4	5	BJTB091202
5	5	2	4	7	5	6	3	4	4	4	5	BJTB091233
5	6	2	4	7	5	6	3	4	4	4	5	BJTB091211
5	6	2	4	7	7	5	2	4	4	4	5	BJTB091302
5	6	2	4	7	7	5	2	4	4	4	5	BJTB091520
5	6	2	4	7	5	5	2	4	4	4	5	BJTB091276
5	6	2	4	7	7	5	4	4	4	4	5	BJTB091538
5	6	2	5	7	7	5	2	4	4	4	5	BJTB091527
5	6	2	3	7	7	5	2	4	4	4	5	BJTB091510
5	6	2	3	7	7	5	4	4	4	4	5	BJTB091694
5	6	2	4	8	7	3	3	4	4	4	5	BJTB091292
5	6	2	4	8	7	6	3	4	4	4	5	BJTB091445
5	6	2	4	8	6	5	3	4	4	4	5	BJTB091366
5	6	2	4	8	6	5	3	4	4	4	5	BJTB091573
5	6	2	4	8	8	5	3	4	4	4	5	BJTB091318
7	6	2	4	8	5	6	3	4	4	4	5	BJTB091204
5	6	3	4	7	8	5	3	4	4	4	5	BJTB091328
5	6	3	4	7	7	5	3	4	4	4	5	BJTB091423
5	6	3	4	7	6	5	3	4	4	4	5	BJTB091389
5	4	3	4	7	4	5	3	4	4	4	5	BJTB091285
5	6	3	4	7	5	5	3	4	4	5	5	BJTB091244
5	6	3	4	7	7	5	3	4	4	5	5	BJTB091493
5	6	3	4	7	7	4	3	4	4	5	5	BJTB091628
5	6	2	4	6	7	5	3	4	4	4	5	BJTB091506
5	6	2	4	6	7	5	3	3	4	4	5	BJTB091552
5	4	2	4	6	8	5	3	4	4	4	5	BJTB091361
5	4	2	4	6	7	5	3	4	4	4	5	BJTB091567
5	4	2	4	6	6	5	3	4	4	4	5	BJTB091382
5	5	2	4	6	7	5	3	4	4	4	5	BJTB091413
5	5	2	4	6	7	5	3	4	4	4	5	BJTB091600
5	5	2	4	6	7	6	3	4	4	4	5	BJTB091463
5	5	2	4	6	7	4	3	4	4	4	5	BJTB091613
5	5	2	4	6	7	6	3	3	4	4	5	BJTB091454
5	5	2	4	6	6	6	3	4	4	4	5	BJTB091352
5	5	2	3	6	4	5	3	4	4	4	5	BJTB090128
5	6	2	4	7	7	4	4	4	4	3	5	BJTB091587
5	6	2	4	6	7	4	3	4	4	3	5	BJTB091855
5	6	2	4	7	7	6	3	4	4	3	5	BJTB091478
5	6	2	4	7	7	5	3	4	4	3	5	BJTB091693
5	6	2	4	7	7	3	3	3	4	3	5	BJTB091438
5	6	2	4	7	7	3	3	4	4	3	5	BJTB091594
5	6	2	4	7	7	6	3	3	4	5	5	BJTB091203
5	6	2	4	7	7	6	3	4	4	5	1	BJTB091466
4	6	2	4	6	7	4	3	4	4	4	5	BJTB091607
4	6	2	4	6	7	4	3	4	4	4	5	BJTB091854
4	6	2	4	7	7	4	3	4	4	4	5	BJTB091434
4	6	2	4	7	7	4	3	4	4	4	5	BJTB091625
4	6	2	4	7	7	4	3	3	4	4	5	BJTB091623
4	6	2	4	7	6	4	3	4	4	4	5	BJTB091637
4	6	2	3	7	6	4	3	4	4	4	5	BJTB091641
1	6	2	4	7	4	4	3	4	4	4	5	BJTB091514
4	5	2	0	7	7	4	3	4	4	4	5	BJTB090489
4	6	2	4	7	8	5	3	4	4	4	5	BJTB091327
4	6	2	4	7	8	3	3	4	4	4	5	BJTB091332
4	6	2	4	7	8	4	3	4	4	4	5	BJTB091616
8	6	2	4	7	8	5	3	4	4	4	5	BJTB091336
3	1	2	4	7	8	5	3	4	4	4	5	BJTB091363
6	6	2	4	7	7	4	3	4	4	7	5	BJTB091339
6	6	2	3	7	7	4	3	4	4	4	5	BJTB091621
6	6	2	2	7	7	4	3	4	4	5	5	BJTB091614
4	6	2	2	7	7	5	3	3	4	4	5	BJTB091489
4	6	2	2	7	7	5	3	4	4	4	5	BJTB091512
4	6	2	3	7	7	5	3	4	4	7	5	BJTB091518
4	6	2	4	7	7	5	3	4	4	5	5	BJTB091349
4	6	2	4	7	7	5	3	4	1	5	5	BJTB091484
4	6	2	4	7	7	5	3	4	2	4	5	BJTB091700
4	6	4	4	7	7	5	3	4	4	4	5	BJTB091401
5	6	4	4	7	7	5	3	4	4	4	5	BJTB091485
4	7	4	4	7	7	5	3	3	4	4	5	BJTB091486
5	6	2	3	7	7	3	3	4	4	4	5	BJTB091303
5	6	2	3	7	7	6	3	4	4	4	5	BJTB091455
5	6	2	3	7	7	6	3	3	4	4	5	BJTB091476
5	6	2	3	7	7	6	4	4	4	4	5	BJTB091435
5	6	2	3	6	7	6	4	4	4	4	5	BJTB091446
5	6	2	3	6	7	6	3	4	4	4	5	BJTB091451
5	6	2	3	5	7	5	3	4	4	4	5	BJTB091528
5	5	2	4	7	8	5	2	4	4	4	5	BJTB091293
5	5	2	4	7	7	5	2	4	4	4	5	BJTB091532
5	5	2	4	7	7	5	5	4	4	4	5	BJTB091304
5	5	2	4	7	8	5	4	4	4	4	5	BJTB091358
4	5	2	4	7	8	5	2	4	4	4	5	BJTB091322
4	5	2	4	7	7	5	4	4	4	4	5	BJTB091410
4	5	2	4	7	7	3	4	4	4	4	5	BJTB091431
4	5	2	4	7	7	5	3	4	4	4	5	BJTB091531
4	5	4	4	7	6	5	3	4	4	4	5	BJTB091278
4	5	2	4	7	6	5	3	4	4	4	5	BJTB091564
4	5	2	4	8	6	5	3	4	4	4	5	BJTB091360
4	5	2	4	7	6	5	3	2	4	4	5	BJTB091578
4	5	2	4	7	5	2	3	4	4	4	5	BJTB091195
4	5	2	4	7	5	5	3	4	4	4	5	BJTB091214
4	5	0	4	7	5	5	3	4	4	4	5	BJTB091243
4	5	2	4	7	6	5	4	4	4	4	5	BJTB091541
3	5	2	4	7	6	5	4	4	4	4	5	BJTB091550
7	4	2	4	7	6	5	4	4	4	4	5	BJTB091300
4	7	2	3	7	6	5	4	4	4	4	5	BJTB091685
4	6	2	3	7	6	5	4	4	4	4	5	BJTB091686
5	5	2	3	7	6	5	1	4	4	4	5	BJTB091517
5	5	2	4	7	6	5	1	4	4	4	5	BJTB091549
5	5	2	3	7	6	5	6	4	4	4	5	BJTB091679
5	5	2	3	7	6	5	1	3	4	4	5	BJTB091681
5	5	2	3	7	8	5	3	4	4	4	5	BJTB091403
5	5	2	3	7	6	5	3	4	4	4	5	BJTB091682
5	5	3	3	7	9	5	3	4	4	4	5	BJTB091333
5	4	2	3	7	7	5	4	4	4	4	5	BJTB091194
5	4	2	3	7	7	5	3	4	4	4	5	BJTB091574
5	5	2	3	7	7	5	3	4	3	4	5	BJTB091698
5	6	2	3	7	6	5	3	4	4	4	5	BJTB091500
5	6	2	3	7	6	5	3	5	4	4	5	BJTB091577
5	6	4	3	7	6	5	3	3	4	4	5	BJTB091687
5	6	2	3	7	8	5	3	2	4	4	5	BJTB091319
8	5	4	3	7	6	5	3	4	4	4	5	BJTB091542
5	6	2	3	7	6	3	3	4	4	4	5	BJTB091667
5	6	2	3	7	6	3	3	4	4	4	5	BJTB091670
5	6	2	3	7	6	3	3	4	4	4	5	BJTB091671
5	5	2	3	7	6	3	3	4	4	4	5	BJTB091660
5	5	2	3	7	6	3	3	4	4	4	5	BJTB091673
5	5	2	3	7	6	3	3	4	4	4	5	BJTB091678
5	20	2	3	7	6	3	3	4	4	4	5	BJTB091661
5	6	2	3	6	6	3	3	4	4	4	5	BJTB091862
4	6	2	2	7	6	3	3	4	4	4	5	BJTB091662
5	6	2	2	7	6	3	3	4	4	4	5	BJTB091672
5	5	6	4	7	6	3	3	4	4	4	5	BJTB091409
5	5	2	4	7	6	3	3	4	4	4	5	BJTB091657
5	3	2	4	7	6	3	3	4	4	4	5	BJTB091555
5	4	2	3	7	6	9	3	4	4	4	5	BJTB091393
5	5	2	6	2	6	3	3	4	4	4	5	BJTB091947
5	4	2	4	7	6	3	3	4	4	4	6	BJTB091655
5	5	3	4	7	6	5	3	4	4	4	5	BJTB091378
5	5	3	4	7	6	4	3	4	4	4	5	BJTB091653
5	5	2	2	7	6	4	3	4	4	4	5	BJTB090203
5	5	2	0	7	6	4	3	4	4	4	5	BJTB090498
5	5	2	4	7	6	4	3	4	4	4	5	BJTB091396
5	4	2	4	7	6	4	3	4	4	5	5	BJTB091642
5	5	2	3	7	6	8	3	4	5	4	5	BJTB091392
5	6	2	4	6	6	5	4	4	4	4	5	BJTB091356
5	6	2	4	8	6	5	4	4	4	3	5	BJTB091499
5	6	2	4	6	6	6	1	4	4	4	5	BJTB091390
5	6	2	4	7	5	4	4	4	4	4	5	BJTB091273
5	6	2	4	7	6	4	4	4	4	4	5	BJTB091611
1	6	2	4	7	6	4	4	4	4	4	5	BJTB091565
5	6	2	4	7	6	4	2	4	4	4	5	BJTB091644
7	6	7	3	7	5	5	3	4	4	4	5	BJTB091213
3	6	1	3	7	7	5	3	4	4	4	5	BJTB091508
9	6	1	4	7	7	6	3	4	4	4	5	BJTB091448
4	5	2	4	7	7	6	3	3	4	4	5	BJTB091464
4	5	2	4	7	7	5	3	3	4	4	5	BJTB091599
5	5	2	4	7	7	5	3	3	4	4	5	BJTB091488
4	5	2	4	7	7	5	3	3	4	4	1	BJTB091597
5	5	2	4	7	7	5	1	3	2	4	5	BJTB091602
6	5	2	4	7	7	6	3	3	2	4	5	BJTB091474
6	3	2	4	7	7	6	3	3	2	4	5	BJTB091480
1	5	2	4	7	8	6	3	3	3	4	5	BJTB091295
5	6	2	4	7	6	5	3	3	4	3	5	BJTB091345
5	5	2	4	7	6	5	3	3	4	3	5	BJTB091537
5	5	2	4	7	6	4	3	3	4	2	5	BJTB091654
5	5	2	4	7	5	5	3	3	4	5	5	BJTB091241
6	5	2	4	7	8	5	3	3	4	3	5	BJTB091326
5	5	2	4	7	8	3	3	3	4	4	5	BJTB091297
5	5	2	4	7	8	5	3	3	4	4	5	BJTB091324
5	3	2	4	7	8	4	3	3	4	4	5	BJTB091337
5	7	2	4	7	5	4	3	3	4	4	5	BJTB091270
3	7	2	4	7	7	3	3	3	4	4	5	BJTB090561
3	2	2	4	7	6	4	3	3	4	4	5	BJTB091603
3	5	4	4	7	4	5	3	3	4	4	5	BJTB091232
6	3	2	4	7	4	5	3	3	3	4	5	BJTB091287
5	7	2	0	7	3	3	3	4	4	4	5	BJTB090478
5	7	0	3	7	7	3	3	4	4	4	5	BJTB091873
5	7	2	4	6	8	3	1	4	4	4	5	BJTB091342
5	4	2	4	7	5	5	4	3	4	4	5	BJTB091231
5	4	2	4	7	7	5	4	3	4	4	5	BJTB091606
5	4	2	4	4	5	8	2	3	4	4	5	BJTB090902
5	3	2	4	3	5	4	4	3	4	4	5	BJTB090121
5	6	2	4	5	5	9	3	4	4	5	5	BJTB090916
5	6	1	4	6	5	5	3	4	4	4	5	BJTB091291
5	8	2	5	6	5	2	3	4	4	5	5	BJTB090135
5	6	2	0	6	5	4	3	4	4	5	5	BJTB090467
5	5	2	4	7	5	6	1	4	4	5	5	BJTB091201
5	6	6	4	6	7	3	3	3	4	4	5	BJTB091877
4	6	6	4	7	6	3	3	4	4	4	5	BJTB091659
4	6	2	4	6	6	3	2	4	4	4	5	BJTB091864
4	6	2	3	7	6	3	3	4	4	3	5	BJTB091674
5	5	4	4	7	6	3	3	4	4	7	5	BJTB091663
5	4	4	3	7	6	3	3	4	4	5	5	BJTB091677
4	4	4	1	7	6	3	3	4	4	4	5	BJTB091676
1	5	2	2	3	7	4	3	4	3	4	5	BJTB091852
5	5	4	2	6	7	4	3	4	4	4	5	BJTB091874
5	3	2	4	6	6	4	3	4	4	4	5	BJTB091638
5	3	2	4	6	6	4	3	4	2	4	5	BJTB091859
5	6	2	3	6	6	4	3	4	3	4	5	BJTB091861
5	5	2	2	6	6	4	2	4	2	4	5	BJTB091853
5	5	2	3	4	5	4	5	4	4	4	5	BJTB090245
5	5	2	3	4	5	4	5	4	4	4	5	BJTB090256
5	5	2	3	4	5	4	3	4	4	4	5	BJTB090130
5	5	2	3	4	5	4	6	4	4	4	5	BJTB090243
4	5	2	3	4	5	4	5	4	4	4	5	BJTB090242
5	5	2	7	4	5	4	3	4	4	4	5	BJTB090758
5	5	2	3	4	5	5	3	4	4	4	5	BJTB091019
5	5	2	3	4	5	5	4	4	4	4	5	BJTB091030
5	5	2	3	2	5	4	2	4	4	4	5	BJTB090854
5	5	2	5	4	5	3	5	4	4	4	5	BJTB091016
5	5	2	3	4	5	2	5	4	2	4	5	BJTB090260
9	5	2	3	4	5	4	5	4	4	5	5	BJTB090241
3	6	2	3	4	5	4	5	3	4	4	5	BJTB090221
5	6	2	3	4	5	4	5	3	4	4	5	BJTB090240
5	6	2	3	4	3	4	5	4	4	4	5	BJTB090228
5	6	2	3	4	3	4	5	4	4	4	5	BJTB090244
5	6	2	3	4	4	4	5	4	4	4	5	BJTB090246
5	6	2	3	4	5	4	5	4	4	4	5	BJTB090259
5	6	2	3	3	3	4	5	2	4	4	5	BJTB090324
5	6	2	3	7	6	4	5	4	4	4	5	BJTB090334
5	6	2	3	3	6	4	5	4	4	4	5	BJTB090342
5	5	2	3	7	6	4	5	4	4	4	5	BJTB090335
5	6	2	2	6	5	4	4	2	4	4	5	BJTB090017
5	6	2	2	5	5	4	6	4	4	4	5	BJTB090238
5	6	2	2	4	5	4	5	4	4	4	5	BJTB090239
5	6	2	2	3	5	4	5	4	4	4	5	BJTB090348
5	6	2	2	3	4	4	5	4	4	4	5	BJTB090329
5	6	2	4	3	5	2	5	4	4	4	5	BJTB090266
5	6	2	4	3	5	4	5	4	4	4	5	BJTB090331
5	6	2	2	3	5	4	5	3	4	4	5	BJTB090247
5	6	2	0	3	5	4	5	3	4	4	5	BJTB090464
5	5	2	2	3	5	3	5	4	4	4	5	BJTB090090
5	6	2	2	3	5	3	5	4	4	4	5	BJTB090097
5	6	2	2	3	5	3	5	4	4	4	6	BJTB090093
5	6	2	3	3	5	2	5	4	4	4	5	BJTB090253
5	6	2	3	3	5	2	5	4	4	4	5	BJTB090314
5	6	2	3	3	5	2	5	4	4	4	5	BJTB090316
5	6	2	3	3	5	2	5	4	4	4	5	BJTB090572
5	6	2	3	7	5	2	5	4	4	4	5	BJTB090315
5	6	2	3	4	5	2	5	4	4	4	5	BJTB090258
5	6	2	3	3	5	2	3	4	4	4	5	BJTB090580
5	6	2	3	3	5	2	3	4	4	4	5	BJTB090707
5	6	2	3	3	5	2	3	4	4	4	5	BJTB090714
5	6	2	3	3	5	2	3	4	4	4	5	BJTB091767
5	6	2	3	3	5	2	3	4	4	4	3	BJTB091807
5	6	2	3	7	5	2	3	4	4	4	5	BJTB090576
5	6	2	3	7	5	2	3	2	4	4	5	BJTB091830
6	6	2	3	3	5	2	1	4	4	4	5	BJTB091933
5	6	2	3	2	5	2	5	4	4	4	5	BJTB090719
5	6	2	3	2	5	3	3	4	4	4	5	BJTB091774
5	6	2	3	2	7	4	3	4	4	4	5	BJTB090681
5	6	2	3	2	5	4	3	4	4	4	5	BJTB091897
5	4	2	3	2	5	2	3	4	4	4	5	BJTB091768
5	4	2	3	2	5	4	3	4	4	4	5	BJTB091829
5	7	2	3	3	5	4	5	4	4	4	5	BJTB090250
5	7	2	3	3	5	4	5	3	4	4	5	BJTB090340
5	3	2	3	3	5	4	5	4	4	4	5	BJTB090320
5	2	2	3	3	5	4	3	4	4	4	5	BJTB090041
5	2	2	3	3	5	4	5	4	4	4	5	BJTB090328
5	7	2	3	3	5	4	3	4	4	4	5	BJTB090112
5	7	2	3	3	5	4	3	4	2	4	5	BJTB090640
5	7	2	3	3	5	4	6	4	4	4	5	BJTB090336
5	3	2	3	3	5	4	3	4	6	4	5	BJTB090636
5	4	2	3	3	5	4	5	4	4	4	5	BJTB090323
5	4	2	3	3	3	4	5	4	4	4	5	BJTB090349
5	4	2	3	3	3	4	3	4	4	4	5	BJTB090670
5	4	2	3	3	5	4	2	4	4	4	5	BJTB091764
5	5	2	3	3	5	4	3	1	4	4	5	BJTB090356
5	5	2	3	3	5	4	3	1	4	4	5	BJTB091762
5	5	2	3	3	5	4	3	5	4	4	5	BJTB091806
5	5	2	3	3	5	4	4	4	4	4	5	BJTB091766
5	5	2	3	3	5	4	4	4	4	4	5	BJTB091808
5	5	2	3	3	5	4	3	4	4	4	5	BJTB090026
5	5	2	3	3	5	4	3	4	4	4	5	BJTB090114
5	5	2	3	3	5	4	3	4	4	4	5	BJTB090644
5	5	2	3	3	5	4	3	4	4	4	5	BJTB091765
5	5	2	3	3	5	4	3	4	4	4	5	BJTB091781
5	5	2	3	3	5	4	3	4	4	4	5	BJTB091785
5	5	2	3	3	5	4	3	4	4	4	5	BJTB091796
5	5	2	3	3	5	4	5	4	4	4	5	BJTB090341
5	5	2	3	6	5	4	3	4	4	4	5	BJTB090031
5	5	2	3	3	5	4	3	4	4	2	5	BJTB090327
5	5	2	3	3	5	4	3	4	4	5	5	BJTB090027
5	5	2	3	3	5	4	4	4	4	5	5	BJTB091791
5	5	2	3	3	6	4	3	4	4	4	5	BJTB090202
5	5	2	3	3	6	4	3	4	4	4	5	BJTB090210
5	5	2	3	3	6	4	3	4	4	4	5	BJTB090215
5	5	2	3	6	6	4	3	4	4	4	5	BJTB091866
5	5	2	3	3	6	4	4	4	4	4	5	BJTB090201
5	5	2	3	3	6	4	3	2	4	4	5	BJTB090190
5	5	2	3	7	5	4	3	4	4	4	5	BJTB090693
5	5	2	3	7	5	4	3	4	4	4	5	BJTB091703
5	6	2	3	7	5	4	3	4	4	4	5	BJTB090696
5	8	2	3	7	5	4	3	4	4	4	5	BJTB090564
5	5	2	3	7	3	4	3	4	4	4	5	BJTB090211
5	6	2	3	3	5	4	3	4	4	6	5	BJTB091790
5	6	2	3	3	5	4	3	4	4	3	5	BJTB091804
5	6	4	3	3	5	4	3	4	4	4	5	BJTB091802
5	6	4	3	3	5	4	3	4	4	4	5	BJTB091900
5	6	2	3	3	5	4	3	4	4	4	5	BJTB090039
5	6	2	3	3	5	4	3	4	4	4	5	BJTB090108
5	6	2	3	3	5	4	3	4	4	4	5	BJTB090637
5	6	2	3	3	5	4	3	4	4	4	5	BJTB090686
5	6	2	3	3	5	4	3	4	4	4	5	BJTB091773
5	6	2	3	3	5	4	3	4	4	4	5	BJTB091894
5	6	2	3	3	5	4	3	4	4	4	5	BJTB091901
5	6	2	3	3	5	4	3	4	4	4	5	BJTB091911
5	6	3	3	3	5	4	3	4	4	4	5	BJTB091914
3	6	2	3	3	5	4	3	4	4	4	5	BJTB090860
6	6	2	3	3	5	4	3	4	4	4	5	BJTB091893
5	6	2	3	3	5	4	2	4	4	4	5	BJTB090650
5	6	2	3	3	5	4	2	4	4	4	5	BJTB090683
5	6	2	3	3	5	4	5	4	4	4	5	BJTB090248
5	6	2	3	3	5	4	5	4	4	4	5	BJTB090251
5	6	2	3	3	5	4	4	4	4	4	5	BJTB091904
5	6	2	3	3	5	4	3	4	4	5	5	BJTB090855
5	6	2	3	3	5	4	3	4	4	5	5	BJTB091815
5	6	2	3	3	6	4	3	4	4	5	5	BJTB091954
3	6	2	3	3	5	4	3	4	4	5	5	BJTB091823
5	6	2	3	3	4	4	3	4	4	4	5	BJTB091786
5	6	2	3	3	4	4	3	4	4	4	5	BJTB091912
5	6	2	3	3	6	4	3	4	4	4	5	BJTB090196
5	6	2	3	3	6	4	3	4	4	4	5	BJTB090208
5	6	2	3	3	6	4	3	4	4	4	5	BJTB090212
5	6	2	3	3	6	4	3	4	4	4	5	BJTB090217
5	6	2	3	3	6	4	3	4	4	4	5	BJTB091955
5	6	2	0	3	6	4	3	4	4	4	5	BJTB090495
5	6	2	3	7	6	4	3	4	4	4	5	BJTB090206
5	6	2	3	7	6	4	3	4	4	4	5	BJTB090214
5	6	2	3	7	6	4	3	4	4	4	5	BJTB091640
5	6	2	3	7	6	4	3	4	4	4	5	BJTB091865
5	6	2	3	7	7	4	3	4	4	4	5	BJTB091882
5	6	2	3	4	6	4	3	4	4	4	5	BJTB090205
5	6	2	3	4	5	4	3	4	4	4	5	BJTB090977
5	6	2	3	1	5	4	3	4	4	4	5	BJTB090697
4	6	2	3	3	5	3	3	4	4	4	5	BJTB091834
4	6	2	3	3	5	3	3	4	4	4	5	BJTB091939
5	6	2	3	3	5	3	3	4	4	4	5	BJTB090665
5	6	2	3	3	5	3	3	4	4	4	5	BJTB090669
5	6	2	3	3	5	3	3	4	4	4	5	BJTB090700
5	6	2	3	3	5	3	3	4	4	4	5	BJTB090800
5	6	2	3	3	5	3	3	4	4	4	5	BJTB091798
5	6	2	3	3	5	3	3	4	4	4	5	BJTB091816
5	6	2	3	3	5	3	3	4	4	4	5	BJTB091819
5	6	2	3	3	5	3	3	4	4	4	5	BJTB091839
5	6	2	3	3	5	3	3	4	4	4	5	BJTB091907
5	6	2	3	3	5	3	3	4	4	4	5	BJTB091940
6	6	2	3	3	5	3	3	4	4	4	5	BJTB091908
5	6	2	3	3	5	3	6	4	4	4	5	BJTB091941
5	6	2	3	3	5	3	3	4	7	4	5	BJTB090634
5	8	2	3	3	5	3	3	4	4	4	5	BJTB091794
[illegible]	[illegible]	[illegible]	[illegible]	[illegible]	[illegible]	[illegible]	[illegible]	[illegible]	[illegible]	[illegible]	[illegible]	[illegible]

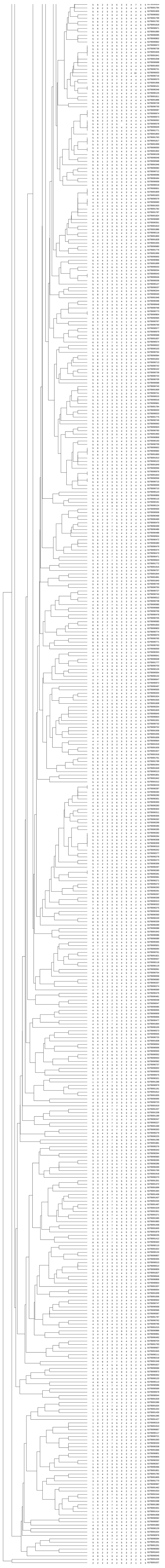

Supplement: Figure S2 — The VNTR cluster of isolate strains collected in various districts from Beijing 2009 by BioNumerics. (PDF) [file pone.0106787.s002.pdf]
